# Foliar spectral signatures reveal adaptive divergence in live oaks (*Quercus* section *Virentes*) across species and environmental niches

**DOI:** 10.1111/nph.70424

**Published:** 2025-09-03

**Authors:** Mariana S. Hernández‐Leal, J. Antonio Guzmán Q., Antonio González‐Rodríguez, Jeannine Cavender‐Bares

**Affiliations:** ^1^ Department of Organismic and Evolutionary Biology Harvard University 22 Divinity Avenue Cambridge MA 02138 USA; ^2^ Instituto de Investigaciones en Ecosistemas y Sustentabilidad Universidad Nacional Autónoma de México Antigua Carretera a Pátzcuaro No. 8701 Morelia 58190 México

**Keywords:** adaptive divergence, isolation‐by‐environment, leaf reflectance spectra, phenotypic plasticity, spectroscopic trait prediction

## Abstract

- Genomic tools have advanced our understanding of species and population structure, but distinguishing neutral from adaptive evolution remains challenging due to limited methods for measuring a broad spectrum of phenotypic traits.
- We used spectroscopic data from preserved leaves to test for adaptive divergence among populations of live oaks (*Quercus* section *Virentes*), a monophyletic group of seven species that diversified under sympatric, parapatric, and allopatric speciation. We used 427 individuals to test for isolation‐by‐distance (IBD) and isolation‐by‐environment (IBE), as well as the influences of selection and phylogenetic inertia on traits. Finally, we examined how phylogenetic signals are distributed across their foliar reflectance spectra.
- Partial redundancy analyses revealed that IBE explains more phenotypic variation than IBD among sympatric species, particularly in certain spectral regions and traits derived from spectra. Phylogenetic generalized least squares models show that environmental variables – including minimum temperature of the coldest month and annual precipitation – predict traits related to stress tolerance across climatic gradients, such as lignin concentration and anthocyanin levels.
- These results demonstrate that foliar reflectance spectra can be used to capture adaptive differentiation and evolutionary history across scales, offering a powerful, nondestructive tool for linking phenotype, environment, and evolutionary processes in long‐lived plant lineages.

## Resumen

- El uso de herramientas genómicas han ampliado significativamente nuestra comprensión de la estructura de especies y poblaciones, pero diferenciar la evolución neutra de la adaptativa sigue siendo un reto debido a la escasez de métodos para evaluar un espectro amplio de rasgos fenotípicos..
- Utilizamos datos espectroscópicos de hojas herborizadas para evaluar la divergencia adaptativa entre poblaciones de encinos del grupo Quercus sección Virentes, un linaje monofilético compuesto por siete especies que se diversificaron mediante especiación simpátrica, parapátrica y alopátrica. Analizamos 427 individuos para probar los patrones de aislamiento por distancia (IBD) y aislamiento por ambiente (IBE), así como la influencia de la selección natural y la inercia filogenética sobre los rasgos fenotipicos. Finalmente, examinamos cómo se distribuyen las señales filogenéticas a lo largo del espectro de reflectancia foliar.
- Los análisis de redundancia parcial (pRDA) revelaron que el aislamiento por ambiente (IBE) explica una mayor proporción de la variación fenotípica que el aislamiento por distancia (IBD) entre especies simpátricas, particularmente en ciertas regiones del espectro y en rasgos derivados de este. Los modelos de mínimos cuadrados generalizados filogenéticos (PGLS) mostraron que variables ambientales—incluyendo la temperatura mínima del mes más frío y la precipitación anual—predicen rasgos asociados con la tolerancia al estrés a lo largo de gradientes climáticos, como el contenido de lignina y los niveles de antocianinas.
- Nuestros resultados muestran que los espectros de reflectancia foliar permiten capturar tanto la diferenciación adaptativa como la historia evolutiva a diferentes escalas, constituyendo una herramienta poderosa y no destructiva para vincular el fenotipo, el ambiente y los procesos evolutivos en linajes de plantas longevas.

## Introduction

In heterogeneous landscapes, selection processes act on populations, generating genetic differentiation and local adaptation, while gene flow – a neutral evolutionary force – limits divergence between populations (Haldane, 1948; Slatkin, 1987; Manel *et al*., 2003; Storfer *et al*., 2007). When gene flow is reduced, genetic drift plays a more significant role, especially in small and isolated populations. Thus, biodiversity arises from both adaptive and nonadaptive processes, and understanding the spatial and temporal scales at which these processes operate is one of the main challenges in evolutionary biology (Bernatchez, 2016; Wellenreuther & Hansson, 2016; Luikart *et al*., 2003).

Advances in genomics have helped clarify the relationships between genetic diversity, spatial structure, and environmental factors (e.g. Hipp *et al*., 2020; Deschepper *et al*., 2017; Kanaka *et al*., 2023). However, genomic data alone are not sufficient to explain phenotypic diversity, which arises through a combination of genetic variation, natural selection, and plastic responses to the environment (Wood *et al*., 2014; Miner *et al*., 2015; Svensson *et al*., 2021). Phenotypes are subject to selection and influence an organism's performance in different environments, driving diversification. By contrast, the genetic structure of neutral genes mainly reflects demographic processes such as genetic drift and changes in effective population size, which are the result of historical biotic and abiotic conditions throughout a species' evolutionary history (Leinonen *et al*., 2013). By comparing the degree of divergence between neutral markers and quantitative traits, it is possible to test for selection to achieve a broader perspective on how populations respond to environmental change (Mackay *et al*., 2009; Hill & Kirkpatrick, 2010). Leaf reflectance spectra provide a new opportunity to describe plant phenotypes and to test selection in comparison with neutral genetic variation. A previous study has shown tight coupling between leaf or canopy optical properties of plants and their evolutionary relationships (Asner & Martin, 2011; Cavender‐Bares *et al*., 2016; Meireles *et al*., 2020; Anderegg, 2023). In recent decades, the use of leaf spectroscopy to quantify biological diversity as a noninvasive method has been intensified, providing rapid and reliable information on leaf optical properties (Asner & Martin, 2016; Cavender‐Bares *et al*., 2016; Meireles *et al*., 2020). Leaf optical properties, such as reflectance, transmittance, and absorbance, are determined by structural and biochemical components that reflect the energy acquisition and resource allocation of plants (Ustin & Gamon, 2010; Cavender‐Bares *et al*., 2017; Kothari & Schweiger, 2022). Spectral phenotypes have been demonstrated to evolve like quantitative traits, with some regions under strong selection and others shaped by neutral processes (Meireles *et al*., 2020).

**Fig. 1:**
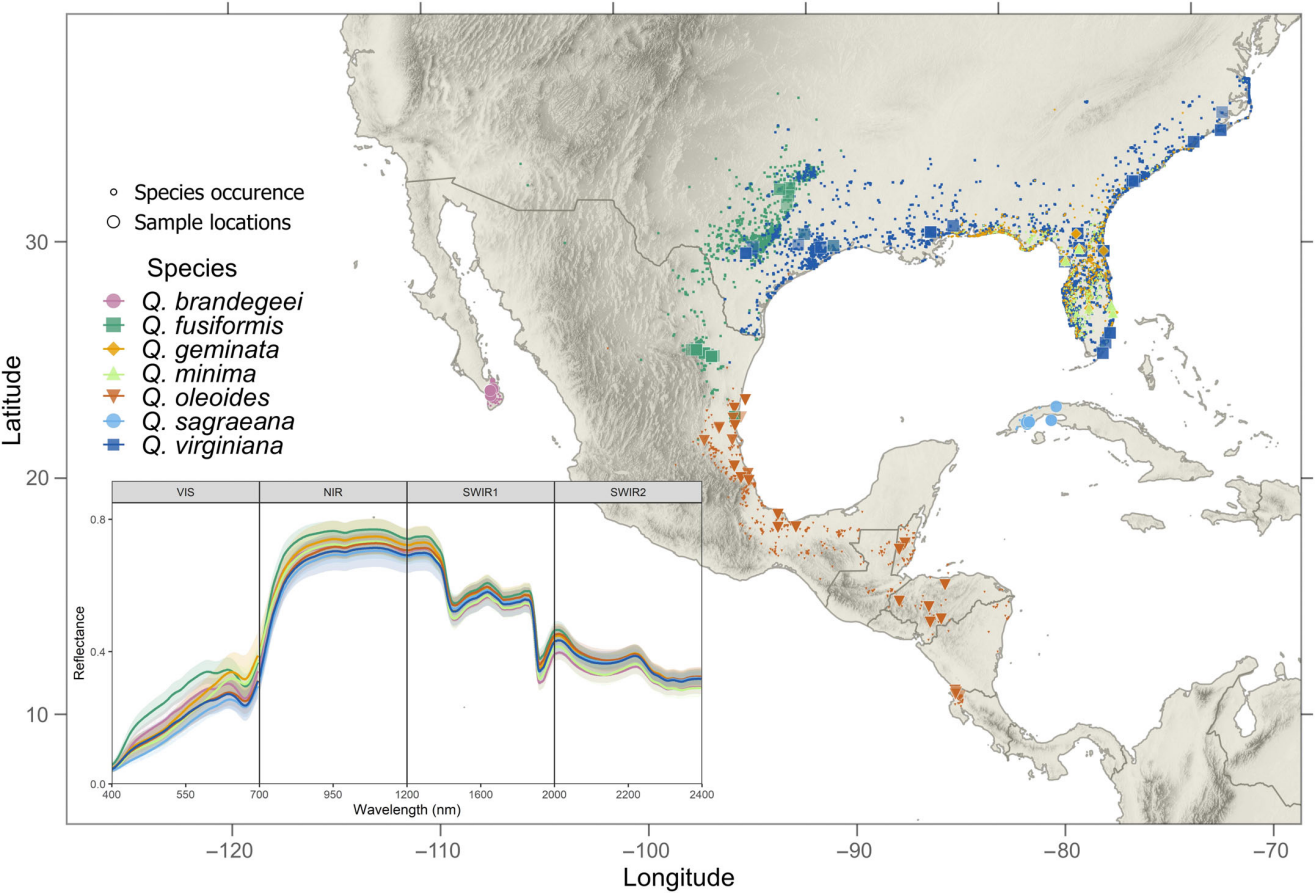
Species occurrence and sample locations of seven species of *Quercus* section *Virentes*. The lower‐left panel represents the mean leaf spectral reflectance of dried, pressed samples (± SD) for each species. Shown are visible, near‐infrared, and first and second short‐wave infrared spectral regions.

This study employs leaf spectroscopy from pressed leaves and existing genetic data to investigate neutral and adaptive evolution in *Quercus* section *Virentes*, a group of seven live oak species spanning from the southeastern United States to Costa Rica, including Baja California Sur and Cuba populations (*Quercus virginiana*, *Quercus geminata*, *Quercus minima*, *Quercus brandegeei*, *Quercus fusiformis*, *Quercus oleoides*, and *Quercus sagraeana*) (Nixon & Muller, 1997; Manos *et al*., 1999; Cavender‐Bares *et al*., 2011). These species inhabit low‐elevation temperate zones with mild winters or seasonally dry tropical climates (Muller, 1961a; Boucher, 1983; Nixon, 1985; Cavender‐Bares *et al*., 2015) (Fig. 1, Supporting Information Fig. S1). Their broad distribution across diverse climates, hydrology, and fire regimes has driven ecological divergence through both allopatric and sympatric processes (Cavender‐Bares, 2019). Phylogenetic studies (Cavender‐Bares *et al*., 2015; Hipp *et al*., 2020) reveal two main clades within the lineage – one with *Q. fusiformis* and *Q. brandegeei*, and another with the other five species. Within the latter clade, *Q. minima*, *Q. virginiana*, and *Q. geminata* coexist sympatrically, while *Q. oleoides* and *Q. sagraeana* are allopatric. Divergence between *Q. virginiana* and *Q. geminata* is maintained by differences in flowering phenology, but *Q. geminata* and *Q. minima* have overlapping flowering periods, promoting introgression (Cavender‐Bares & Pahlich, 2009). Despite indications of genetic similarity based on nuclear microsatellite and chloroplast data, differences in habitat linked to leaf function, including fire dependency and leaf traits, distinguish these species (Kurz & Godfrey, 1962; Cavender‐Bares *et al*., 2004b, 2015). Given the large climatic gradients that live oaks collectively span, natural selection is likely to have shaped leaf functional traits within and across species.

Here, we evaluate the extent to which leaf reflectance spectra from preserved samples can detect phenotypic divergence and adaptive evolution within and among genetic groups at different hierarchical levels, encompassing both inter‐ (phylogenetic) and intraspecific (population‐level) variation. The study has four main objectives. First, we evaluate the role of isolation‐by‐distance (IBD) and isolation‐by‐environment (IBE) (Wright, 1949; Wang & Bradburd, 2014) in driving phenotypic divergence among sympatric species (*Q. virginiana*, *Q. geminata*, and *Q. minima*), with a focus on trait–environment relationships and gene flow. Second, we investigate the association of ecological variables and phenotypic variation among closely related species that have been separated into distinct abiotic environments for millions of years (*Q. fusiformis* vs *Q. brandegeei* and *Q. oleoides* vs *Q. sagraeana*) to assess the relative influence of adaptive trait evolution (selection) and shared evolutionary history (phylogenetic inertia) on functional leaf traits. Third, we test for within‐species adaptive variation leveraging the broad latitudinal and environmental range of *Q. oleoides* – which spans diverse precipitation regimes – to test the ability of spectral data to compare phenotypic divergence among populations to their genetic divergence. In doing so, we extend our investigation of adaptive evolution to a finer genetic scale. Finally, we seek to identify regions of the electromagnetic spectrum (i.e. 400–2500 nm) capable of capturing phylogenetic signals within the section *Virentes* and evaluate how the strength and distribution of this signal vary across the phylogeny. We specifically compare regions of the spectrum that are strongly linked with pigments such as the visible (VIS; i.e. 400–700 nm) region to those mostly influenced by structure in the near‐infrared (NIR; i.e. 700–1200 nm) region and the concentration of chemical compounds in the short‐wave infrared (SWIR; i.e. 1200–2500 nm) region.

We test four hypotheses about adaptive differentiation in ecological niches at different biological and geographic scales within the live oaks based on foliar spectral signatures in relation to neutral molecular markers and phylogenetic information. First, we hypothesize that closely related sympatric species (*Q. virginiana*, *Q. minima*, and *Q. geminata*) exhibit distinct leaf phenotypes that are adaptive to contrasting hydrologic microhabitats, despite gene flow (Cavender‐Bares & Holbrook, 2001), as evidenced by greater leaf phenotypic variation than can be explained by distance alone. Across all live oak populations, we further expect that traits such as leaf mass per area (LMA) anthocyanin and, lignin concentration, along with spectral features tied to leaf structure, will covary with source environmental variation in directions that support the hypothesis of habitat‐driven adaptations. Second, we hypothesize that phenotypic divergence between pairs of closely related allopatric species (*Q. fusiformis* and *Q. brandegeei* or between *Q. oleoides* and *Q. sagraeana*) is a consequence of adaptive evolution associated with ecological divergence enforced by vicariance and limited gene flow. Third, we hypothesize that within a single species (*Q. oleoides*), differentiation in plant phenotypes will be associated with environmental variation among populations in the direction expected based on known functions of plant traits and attributable to adaptive evolution. Finally, we hypothesize that across the *Virentes* phylogeny, spectral bands in the VIS region – in which selection to conserve the photosynthetic machinery is strong – will show less lability and greater phylogenetic signal than in the NIR or SWIR, in which spectral bands are linked to chemical and structural aspects that we expect to be labile and to vary with divergent evolutionary pathways.

## Materials and Methods

### Sample collection

Individual trees of each *Virentes* species (*Quercus virginiana* Mill., *Quercus geminata* Small, *Quercus minima* (Sarg.) Small, *Quercus brandegeei* Goldman, *Quercus fusiformis* Small, *Quercus oleoides* Schltdl. & Cham., and *Quercus sagraeana* Nutt.) were sampled throughout their occurrence ranges and within common gardens between 2003 and 2016 for DNA extraction and leaf phenotyping (Fig. 1). In natural populations, identification of species was based on leaf, bark, and stem height characters following Muller (1961a,b), Kurz & Godfrey (1962) and Nixon & Muller (1997). Multiple leaves from each tree were pressed and stored in a dry cabinet in the laboratory, with representative voucher specimens housed in the University of Minnesota Bell Museum of Natural History. Common garden experiments were established at the University of Minnesota Plant Growth Facilities from seeds collected in natural populations. Leaves from individual saplings from the common gardens were pressed, dried, and stored in a dry cabinet (Table S1). Additional traits, such as freezing and drought tolerance and differences in growth form, further reinforce reproductive isolation (description of environment for each species can be found in Methods S1).

### Neutral genetic variation and genetic structure

We used 11 nuclear simple sequence repeat (nSSR) loci previously employed in assessing neutral genetic variation in *Quercus* (Cavender‐Bares & Pahlich, 2009; Cavender‐Bares *et al*., 2011, 2015; Gugger & Cavender‐Bares, 2013). These markers were selected for their high polymorphism and proven neutrality, which makes them suitable for investigating processes such as genetic drift and gene flow across populations and species (e.g. Liu *et al*., 2005; Gaerke *et al*., 2012; Álvarez *et al*., 2021).

To evaluate genetic structure, we conducted a Bayesian clustering analysis in structure v.2.3.4 (Pritchard *et al*., 2000) using the admixture model and no prior information on sampling location (Hubisz *et al*., 2009). Analyses were performed both at the species level and within *Q. oleoides*, the only species suited for population‐level analysis due to its broad latitudinal distribution and high‐quality sampling across environmental gradients, allowing us to assess intraspecific structure that was not possible for the other species due to more limited and uneven sampling. For both analyses, we used multilocus nSSR data and allowed the number of clusters (*K*) to range from 1 to 9, following previous work (Cavender‐Bares *et al*., 2015). Each structure run was performed independently using an admixture model without location priors, with a burn‐in of 2 × 10^5^ steps followed by 2 × 10^6^ Markov Chain Monte Carlo (MCMC) iterations. The most likely number of clusters (*K*) was determined using the ΔK method (Evanno *et al*., 2005) and visualized with structure harvester v.0.6.94 (Earl & VonHoldt, 2012). We used a threshold of 0.6 to identify individuals with a majority membership in one cluster, rather than to define pure individuals in a strict sense. This lower threshold was selected to reflect the high levels of admixture observed in *Quercus* species and to be inclusive of the actual genetic composition of individuals within populations in a system known for extensive gene flow. We calculated pairwise genetic differentiation between species and genetic groups using *F*_ST_ estimators implemented in the hierfstat package in R (Goudet, 2005). The details of Bayesian clustering are found in Methods S2.

### Dried‐leaf reflectance spectra measurements and preprocessing

Pressed samples are stored at the Harvard University Herbaria. Leaf reflectance was measured on the adaxial surface of three fully expanded leaves in each of the 427 individuals using a leaf clip with an internal light source attached to a high‐spectral resolution field spectroradiometer SVC HR‐1024i (Spectra Vista Corp., Poughkeepsie, NY, USA). Leaf reflectance spectra were corrected for the splice in bands near 990 and 1900 nm using the spectrolab package in R (Meireles *et al*., 2017). Subsequently, spectra were resampled to 3 nm and transformed using continuous wavelet transformation (CWT). CWT was used as a method to isolate and enhance spectral features to improve discrimination among species and phenotypes (White *et al*., 2025). This transformation was computed based on a second‐order derivative of Gaussian using scales 2^2^, 2^4^, and 2^6^. Wavelet scales were then summed for further analyses. Bands at the edge of the spectrometer range (< 400 nm and > 2450 nm) were excluded for further analysis due to low signal‐to‐noise ratios or transformation artifacts. The CWT was performed using the wavCWT function in the wmtsa package of R (Constantine & Percival, 2016). The details on CWT spectra transformation are provided in Methods S3.

### Estimation of leaf traits from spectra

We predicted leaf reflectance spectra functional traits using previous measurements of dried‐leaf reflectance spectra. Specifically, we focused on six leaf structural traits: leaf mass per area (LMA; kg m^−2^), leaf dry thickness (mm), concentrations of cellulose (%), hemicellulose (%), cell solubles (%), and lignin (%). Estimations of leaf traits were performed excluding petioles from unmounted pressed specimens. The concentrations of carbon fractions, including cell solubles (nonstructural carbohydrates, cell contents such as carbohydrates, lipids, pectin, starch, soluble proteins, and nonprotein nitrogen), hemicellulose, cellulose, and lignin (%) were obtained from sequential digestion (ANKOM Fiber Analyzer 200; ANKOM Technology, Macedon, NY, USA).

We used a common partial least squares regression (PLSR) modeling framework to predict leaf traits from dried‐leaf reflectance spectra across the full range (400–2450 nm). This framework involves three main steps: (1) split the data into training and testing datasets; (2) selection of the optimal number of components; and (3) assessment of the models. A detailed description of the employed framework is presented in Methods S4.

### Partial least squares discriminant analysis for species classification

We employed a partial least squares discriminant analysis (PLS‐DA) model to classify dried‐leaf wavelet spectra among the seven species in *Virentes*. Each species was represented by a minimum of 20 samples. The dataset was divided into 70% training (calibration) and 30% testing (validation) subsets based on the species‐genetic group level. We first conducted an iterative PLS‐DA (50 iterations) using the bootstrap method to control sample size; to determine the optimal number of components. After selecting the optimal number of components, a final PLS‐DA model was generated based on 50 iterations. We assessed the classification performance using confusion matrices between species and genetic groups. PLS‐DA modeling was conducted using the caret R package (Kuhn *et al*., 2020).

### Bayesian clustering based on spectrally predicted traits

To compare phenotypic structure, we used the unsupervised Bayesian clustering algorithm geneland v.4.0.9 (Guillot & Santos, 2009). Six leaf traits predicted from reflectance spectra were used in combination with the geographic location of individuals to identify phenotypic clusters. This analysis was performed at two hierarchical levels – among the seven *Virentes* species and among populations of *Q. oleoides*. In order to evaluate trait differentiation potentially shaped by local adaptation at the population level, we performed a separate analysis for *Q. oleoides*, the only species for which sampling depth and environmental coverage allowed robust comparisons among populations. To explore the number of phenotypic clusters, we conducted 11 independent runs with *K* ranging from 1 to 10, using 2 × 10^6^ MCMC iterations and a thinning interval of 1000. We subsequently fixed the value of *K* at 6 for the species‐level analysis and at *K* = 5 for the *Q. oleoides* populations. We ran 20 additional replicates for each *K* and retained those with the highest mean logarithmic posterior probabilities, applying a 10% burn‐in. Each spatial cell was set to *c*. 10 km^2^, and individuals with cluster membership probability < 0.7 were considered admixed. Results were summarized using clumpp v.1.1.2 (Jakobsson & Rosenberg, 2007).

### Comparing phenotypic vs genotypic divergence

Analyzing the relationship between phenotypic and genetic variation provides a means to test for selection, particularly through *P*_ST_–*F*_ST_ comparisons (Merilä & Crnokrak, 2001; McKay & Latta, 2002). We compared nSSR‐derived genetic distances (*F*_ST_) with quantitative trait distances (*P*_ST_) based on six spectrally predicted leaf traits, as well as key wavelengths within three main spectral regions: the VIS (400–700 nm), NIR (700–1200 nm), and SWIR (1200–2500 nm).

For the species‐level analysis, *P*_ST_ and *F*_ST_ values were compared across all seven *Virentes* species (phylogenetic scale). In addition, we conducted a complementary set of *P*_ST_–*F*_ST_ comparisons specifically within *Q. oleoides* (between‐population scale) on six spectrally predicted leaf traits to assess evidence for selection by comparing across genetic and phenotypic variation between population levels. Specifically, we tested for within‐species adaptive differentiation along environmental gradients. Spectral traits were selected based on high variable importance in projection (VIP) scores from PLS‐DA models using continuous wavelet‐transformed reflectance data. Initially, we selected all wavelengths with VIP > 0.8 and then included additional bands with VIP ≥ 0.4 to capture a broader set of discriminant traits (Wold *et al*., 2001). The details on *P*_ST_ calculation using both spectrally predicted traits and raw spectra are provided in Methods S5.

### Spatial and environmental drivers of trait divergence among species

To determine whether trait differentiation among species follows patterns of IBD or IBE, we used redundancy analysis (RDA) to compare genetic, spatial, and phenotypic variation against variation in environmental variables. This comparison provides a means to test whether environmental factors explain phenotypic differences beyond the effects of geographic distance. To select the environmental variables included in the RDA, we first reduced multicollinearity by conducting a variance inflation factor (VIF) analysis and retained only variables with VIF values below a conservative threshold, ensuring independent predictors for the models.

For each pair of species, partial RDA was conducted to partition the explainable phenotypic variation into those attributable to spatial (SPACE), genetic (GEN), environmental factors (ENV), and their combined effect. The full, partial, and joint contributions of SPACE, ENV, and GEN to the explainable phenotypic variations were estimated and tested for significance, and the most influential single explanatory variables were identified (Dataset S1). For more detailed explanation of partial redundancy analysis (pRDA) models, see Methods S6.

### Testing for phylogenetic signal

We used phylogenetic generalized least squares regression (PGLS) to analyze phylogenetic signals in phenotypic traits while simultaneously examining how these traits relate to environmental variation. The analysis was conducted using the phylogenetic tree derived from RADseq data (Cavender‐Bares *et al*., 2015) as a framework, which included 17 samples representing different populations from the seven *Virentes* species. To obtain the variation in phenotypic characters for these populations, we calculated the mean trait values across individuals within each population. Spectral traits were analyzed separately for each region of the spectrum: VIS, NIR, and SWIR, as well as for six key leaf traits derived from leaf spectroscopy. To gain a broader perspective, the same method was also applied to various vegetation indices that characterize features associated with biophysical and chemical properties. The indices selected were Chl Index Red Edge (CI = 750 nm/710 nm) as a descriptor of the Chl concentration (Gitelson *et al*., 2003) and Normalized Difference Water Index (NDWI = (835 nm–1610 nm)/(835 nm + 1610)) as a descriptor of the leaf water content (Quemada *et al*., 2021).

To evaluate the significance of the observed *K* values, we compared them to null distributions generated under two models. First, a white noise (WN) model was used, in which trait values were randomly permuted across the phylogeny's tips 1000 times. Second, a Brownian motion (BM) model simulated trait evolution under BM across the phylogeny 1000 times. *K* values falling below the 95% distribution of the simulated BM values suggest that traits are less phylogenetically structured than expected under BM (Blomberg *et al*., 2003), and the Anthocyanin Reflectance Index (ARI = (1/550 nm)–(1/700 nm)) as a descriptor of the anthocyanins concentration (Li *et al*., 2023). Overall, this approach allowed us to assess the phylogenetic structure and environmental associations for each spectral region, vegetation index, and predicted leaf trait independently, providing insights into how evolutionary and environmental factors shape spectral variation. The most common environmental variables used in pRDA were employed after removing highly correlated variables (based on Pearson's correlation |*r*| ≤ 0.60) to control for multicollinearity for each pairwise species. To evaluate the correlation between environmental variables and the traits of 427 samples, and to complement the information and gain a better understanding of how environmental variables relate to the traits, a Pearson's correlation was performed, as opposed to the PGLS, which relied only on the phylogeny of 17 sequenced individuals and their corresponding population mean leaf trait values. The phylogenetic signal was quantified using Blomberg's *K* to determine the extent to which spectral data were conserved along the phylogeny (Blomberg *et al*., 2003) implemented in phytools (Revell, 2012). Blomberg's *K* measures the degree to which trait variance lies within clades vs among clades as compared to a Brownian expectation. Significance was assessed using 999 tip‐swap randomizations. The package phylosig in R (Revell, 2012) was chosen because it incorporates SE in its calculations and allows flexibility in estimating Blomberg's *K* with different settings. A value of *K* = 0 indicates no phylogenetic signal, while *K* > 1 suggests that closely related species exhibit stronger trait similarities than expected under a BM model of evolution (Blomberg *et al*., 2003).

### Accounting for environmental influence on phenotypes using spectral variation in leaves from a common garden

We assessed the phenotypic differences among species independently of environmental effects by using pressed‐leaf samples from a common garden. To do so, we measured reflectance spectra from pressed leaves from 22 individuals of each of four species (*Q. fusiformis*, *Q. geminata*, *Q. oleoides*, and *Q. virginiana*). These individuals were grown in a common garden in a controlled environment at the Plant Growth Facility on the St Paul campus of the University of Minnesota. Phenotypic distances (*P*_ST_) from spectrally derived trait values were calculated in the same way as for wild populations (VIP wavelengths were not used in this case). All pairwise estimates of *P*_ST_, their confidence intervals (CI), and the comparison with *F*_ST_ genetic distances were calculated using the package pstat in R (Silva & Silva, 2018). *F*_ST_ (and CI) were estimated using the *boot.ppfst* function in the hierstat R package.

## Results

### Neutral genetic variation

Hierarchical analyses of molecular variance under the infinite alleles model (*F*_ST_) indicated that 9.2% (*P* < 0.001) of the neutral genetic variation is found among species, 6.5% (*P* < 0.001) among populations within species, 13.7% (*P* < 0.001) among individuals within populations, and 70.6% (*P* < 0.001) within all the individuals (Table S2a). Pairwise genetic differentiation (*F*_ST_) among species (phylogenetic scale) based on nSSRs was statistically significant (*P* < 0.05) for all species pairs, with *F*_ST_ values ranging from 0.012 to 0.27. The greatest level of genetic differentiation was observed between *Q. brandegeei* and *Q. geminata* (Table S2b). Within *Q. oleoides*, all pairwise genetic differentiation values *F*_ST_ between genetic groups – defined based on Bayesian clustering analyses using structure – were significant (*P* < 0.05). *F*_ST_ values ranged between 0.03 and 0.132, in which the highest values were found between genetic groups 4 vs 1 (Costa Rica vs North Mexico) and genetic groups 4 vs 2 (Costa Rica vs South Mexico; Table S2c).

### Functional leaf traits from spectra

The PLSR models for estimating traits using the internal validation dataset show high‐to‐moderate accuracy and precision. Accuracy, kappa, sensitivity, and specificity using 20 PLSR components results are in Table S3. The best spectrally predicted traits were LMA (*R* ^2^ = 0.88, RMSEP = 17.6), cellulose (*R* ^2^ = 0.72, RMSEP = 3.37), and thickness (*R* ^2^ = 0.65, RMSEP = 0.8; Table S4; Fig. S2). VIP was used to identify which regions of the spectrum were important in predicting leaf traits (Fig. S3). For all spectrally predicted leaf traits, the ranges between 660–680 and 750–780 nm (VIS) (Fig. S3a,c) showed the highest importance in predicting traits. SWIR range was important to predict lignin and cell solubles concentrations (carbohydrates, lipids, pectin, starch, soluble proteins, and nonprotein nitrogen) *c*. 1920–2050 nm (Fig. S3b,d). The NIR and much of the SWIR region (i.e. 1400–1850 nm) were less important for predicting leaf traits.

### Bayesian clustering based on genetic data and phenotypic traits

**Fig. 2:**
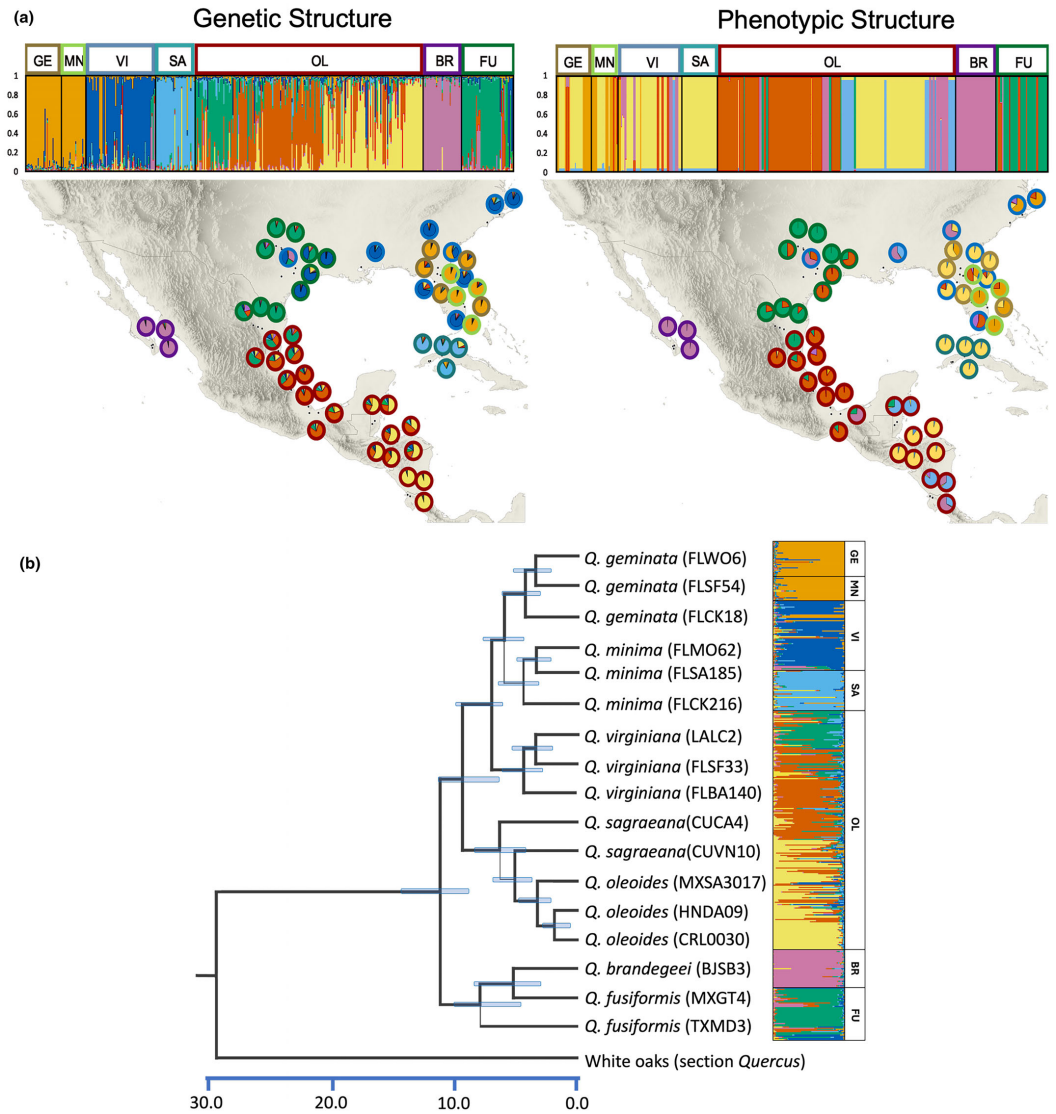
Spatial distribution of genetic and phenotypic variation of seven oak (*Quercus*) species of the section *Virentes*. (a) Seven genetic groups identified by structure from 56 populations. (b) Six phenotypic groups identified by GENELAND using six leaf traits derived from reflectance spectra from the same 56 populations. The percentage assignment to genetic or phenotypic groups is represented at both the individual tree level (upper bar plots) and subpopulation level (pie charts). Colors outlining the pie charts represent species of sample origin. Colors inside the pie charts represent genetic (a) or phenotypic (b) groups. BR, *Quercus brandegeei*; FU, *Quercus fusiformis*; GE, *Quercus geminata*; MN, *Quercus minima*; OL, *Quercus oleoides*; SA, *Quercus sagraeana*; VI, *Quercus virginiana*. (b) Time‐calibrated phylogeny inferred from RADseq data for 17 *Virentes* individuals and three outgroup taxa in beast v.10.X using three priors for node ages based on fossil (data not shown). Line widths indicate support for nodes. Blue bars show the 95% highest posterior density values around each age estimate (Cavender‐Bares, 2015).

Using the nSSRs from 427 individuals, the clustering analysis suggested seven (*K* = 7) most probable genetic groups with a mean log_e_P (*K*) = −3337.23 and *ΔK* = 96.93. This result is like those obtained in Cavender‐Bares *et al*. (2015), indicating that the reduced sample size did not meaningfully change the genetic clustering. However, genetic clusters did not strictly align with species boundaries, particularly among sympatric taxa. For instance, *Q. geminata* and *Q. minima* formed a distinct genetic group (Cluster 1; Fig. 2a), while *Q. virginiana* exhibited notable admixture, consistent with previous findings (e.g. Cavender‐Bares *et al*., 2015). These results reflect the complex evolutionary history of the *Virentes* clade, shaped by recent divergence and gene flow. Using the GENELAND algorithm, the most probable number of clusters obtained based on all spectrally predicted traits (i.e. phenotypes) was *K* = 6. Phenotypic group 2 combines individuals from five different species, showing similar phenotypic characteristics in *Q. geminata*, *Q. minima*, *Q. virginiana*, *Q. sagraeana*, and Central American populations of *Q. oleoides* (Fig. 2b). Clustering within *Q. oleoides* using nSSR data showed four major genetic groups (*K* = 4) with a mean log_e_ *P*(*K*) = −7935.1 and *ΔK* = 13.02. This analysis describes genetic groups divided into major geographical regions: Northeastern Mexico (N. MX), Southeastern Mexico (S. MX.), Central America (BZ and HND), and Costa Rica (CR). Phenotypic clustering using GENELAND showed five distinct phenotypic groups, suggesting differences with respect to genetic groups (Fig. S4).

### Partial least squares discriminant analysis for species classification

**Fig. 3:**
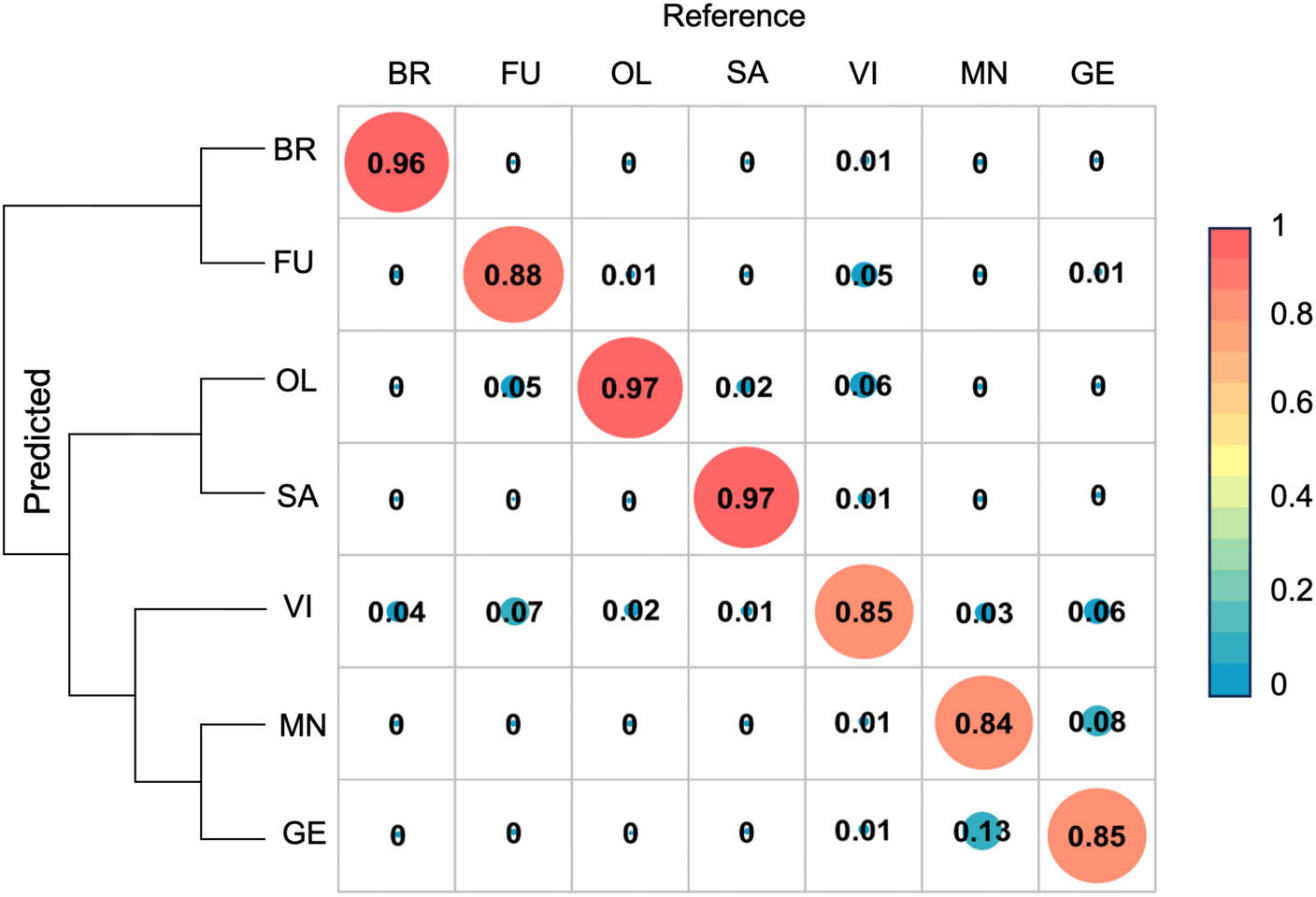
Confusion matrices from partial least squares discriminant analysis for seven *Quercus* species of section *Virentes*. Columns represent the observed identities, while rows indicate the predicted identities and phylogenetic relations among species. Values along the diagonals show the percentage of individuals accurately classified within each group, while values above or below the diagonals show the percentage of misclassification. Left side phylogeny to show species relations. BR, *Quercus brandegeei*; FU, *Quercus fusiformis*; GE, *Quercus geminata*; MN, *Quercus minima*; OL, *Quercus oleoides*; SA, *Quercus sagraeana*; VI, *Quercus virginiana*.

Bayesian clustering structure analysis using the molecular SSR data (Fig. 2a) revealed high rates of admixture between individuals of *Q. geminata* and *Q. minima*, clustering them in the same genetic group. Spectrally derived traits using the unsupervised Bayesian clustering algorithm GENELAND also showed clustering of *Q. geminata* and *Q. minim*a in the same group (Fig. 2b). By contrast, the PLS‐DA classification model using all spectra from pressed leaves was able to discriminate individuals among the seven taxonomically identified species of *Virentes*. This analysis correctly predicted the taxonomic identity of 295 of 302 samples in the training dataset (Fig. 3), discriminating individuals among species that have sympatric distributions with high accuracy. For example, it achieved 85% classification accuracy for *Q. geminata*, 84% for *Q. minima*, and 85% for *Q. virginiana*.

### Comparing phenotypic vs genotypic divergence

Using leaf traits derived from spectra, sympatric species (i.e., *Q. geminata vs Q. virginiana*) showed significant differences in their *P*_ST_ pairwise distances (*c* : *h* ^2^ > 0.25) for almost all traits compared with *F*_ST_, except for cellulose. *Quercus minima* vs *Q. virginiana* had significant differences in their *P*_ST_ pairwise distances for LMA, leaf thickness, cell solubles, and hemicellulose (*c* : *h* ^2^ > 0.25) and cellulose and lignin when (*c* : *h* ^2^ > 0.5). For the pairwise comparison between *Q. geminata* and *Q. minima*, only cellulose showed significantly higher values of *P*_ST_ (0.86) than that of *F*_ST_ (0.011).

**Fig. 4:**
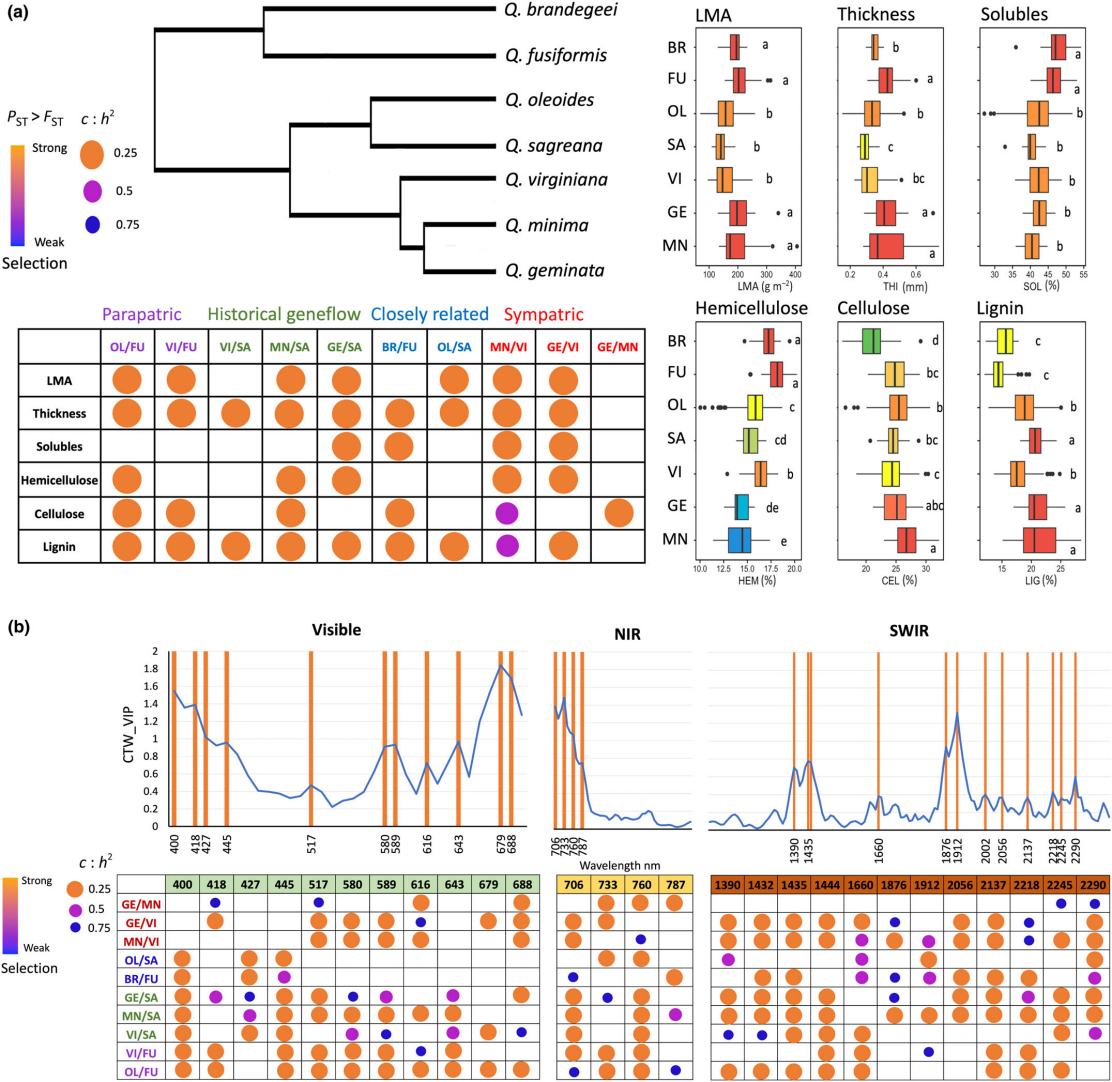
Pairwise *P*_ST_ comparisons: Matrices represent *P*_ST_ plotted as a function of *c*/*h* ^2^ values selected at 0.25 (orange), 0.5 (pink), and 0.75 (blue). The optimal value of *c*/*h* ^2^ at which the lower confidence limit of *P*_ST_ is higher than the upper confidence limit of *F*_ST_. (a) Spectrally predicted traits (CEL, cellulose; HEM, hemicellulose; LIG, lignin; LMA, leaf mass area; SOL, solubles; THI, thickness). Left shows a simplified phylogenetic tree inferred from RADseq data for 17 Virentes individuals using RAXML (Cavender‐Bares *et al*., 2015) with the pairwise comparisons among species that were conducted using the six spectrally predicted traits. Colored pairs represent sister relationships, historical introgression between specie pairs, and/or sympatric geographic associations within the Virentes: red, sympatric sister species; blue, sister but not sympatric species; green, historically introgressing populations; purple, parapatric species with introgression. Right: Box‐and‐whisker plots showing the distribution of each trait for each species. The vertical line represents the median, the box spans the interquartile range (25^th^–75^th^ percentiles), whiskers extend to 1.5× the interquartile range, and points beyond whiskers indicate outliers. Colors indicate Tukey groups, with different letters denoting significant differences (*P* < 0.05). (b) Variable importance of projection (VIP) spectral bands selected within the visible (VIS), near‐infrared (NIR), and short‐wave infrared (SWIR) regions based on importance in discrimination among species using partial least squares discriminant analysis (PLS‐DA), represents plotted VIP values obtained from the PLS‐DA classification model using wavelet spectra: Orange vertical lines represent wavelengths that were used as traits to calculate *P*_ST_ pairwise distances. Matrices are divided into VIS, NIR, and SWIR spectral regions.phylogenetic/geographic relations. BR, *Quercus brandegeei*; FU, *Quercus fusiformis*; GE, *Quercus geminata*; MN, *Quercus minima*; OL, *Quercus oleoides*; SA, *Quercus sagraeana*; VI, *Quercus virginiana*. Only phylogenetically and geographically meaningful pairwise comparisons are shown.

Significant differences between *P*_ST_–*F*_ST_ for sister species *Q. oleoides* and *Q. sagraeana* (*F*_ST_ = 0.073) showed evidence for phenotypic selection on three leaf traits: LMA (*P*_ST_ = 0.8), thickness (*P*_ST_ = 0.88), and lignin (*P*_ST_ = 0.93). Differentiation measures between *Q. brandegeei* and *Q. fusiformis* (*F*_ST_ = 0.153) were also significantly different on four traits: leaf thickness (*P*_ST_ = 0.96), cell solubles (*P*_ST_ = 0.92), cellulose (*P*_ST_ = 0.93), and lignin (*P*_ST_ = 0.9). Fig. 4 shows trait difference for pairwise comparisons among species with *c* : *h* ^2^ values of 0.25, 0.5, and 0.75. The pairwise comparisons among genetic groups of *Q. oleoides* also revealed adaptive signals in leaf thickness and lignin, as evidenced by *P*_ST_ values that significantly differed from *F*_ST_ for all genetic groups, except for CR and Honduras (CR vs HN). Detailed results for all pairwise comparisons can be found in Fig. S5.

Using bands with high importance in discriminating species (i.e. VIP) from the PLS‐DA species classification model, we found that closely related species tended to be more spectrally similar in the visible region than more distantly related species. Given that our main objective is to focus on adaptive differentiation in ecological niches at different biological and geographic scales, we only present pairwise comparison values of phenotypic significance, *P*_ST_ > *F*_ST_, among sympatric species (*Q. geminata*, *Q. virginiana*, and *Q. minima*), allopatric sister species (*Q. fusiformis* and *Q. brandegeei* or *Q. oleoides* and *Q. sagraeana*), and widely distributed species with parapatric populations (*Q. oleoides* and *Q. fusiformis*; *Q. virginiana*) shown in Fig. 4(b). Detailed results for all pairwise comparison values of phenotypic *P*_ST_–*F*_ST_ obtained from spectrally derived traits and their confidence intervals can be found in Fig. S6 and Dataset S2.

### Spatial and environmental drivers of species‐level trait divergence

**Fig. 5:**
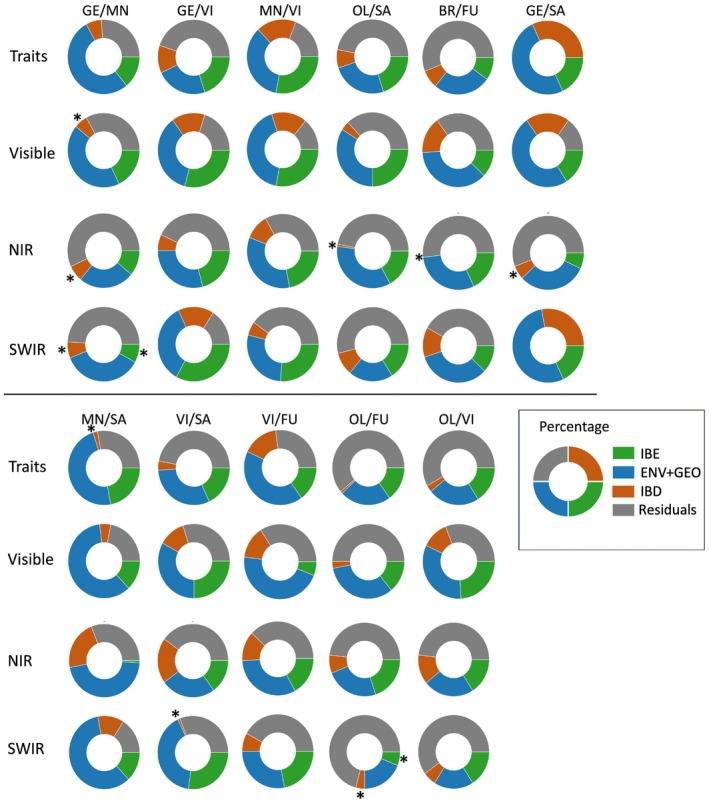
Redundancy analysis of divergence in pairwise *Quercus* section *Virentes* species. Radians represent the percentage of the total variation in spectrally derived traits and spectral variable importance in projection wavelengths in each region visible, near‐infrared, and short‐wave infrared that can be explained by space: Environment: PHENO ~ ENV + Condition (GEO) (green IBE); PHENO ~ GEO + Condition (ENV) (brown IBD) and their interaction PHENO ~ GEO + ENV (blue). Asterisk (*) indicates that the proportion of variance explained (PVE %) is not significant. BR, *Quercus brandegeei*; FU, *Quercus fusiformis*; GE, *Quercus geminata*; MN, *Quercus minima*; OL, *Quercus oleoides*; SA, *Quercus sagraeana*; VI, *Quercus virginiana*. Environmental variables peer individuals are listed in Dataset S1.

We performed multivariate RDAs to attribute explainable variation in phenotypic traits to spatial location, environment, or their joint effect. This analysis integrates two classical models of population structure: IBD and IBE. The results show the percentage of variation explained by environmental variables controlling space PHENO ~ ENV + Condition (GEO), spatial variables controlling environment PHENO ~ GEO + Condition (ENV), and the combination of both (PHENO ~ GEO + ENV) for different species pairs (e.g. *Q. geminata* vs *Q. minima* GE/MI, *Q. geminata* vs *Q. virginiana* GE/VI). Overall, the total variation explained by environment and spatial location varies widely across species pairs, ranging from 20% (OL/SA) to 57% (GE/SA). In most cases, the variation explained by environmental variables (IBE) is greater than that explained solely by spatial factors (IBD), suggesting that environmental conditions play a more significant role than spatial variation in determining spectral traits. This is particularly evident in pairs, including GE/MI (18% vs 6.5%) in the visible range and GE/VI (33% vs 16%) in the SWIR region. However, in certain cases (e.g. GE/SA), spatial factors explain a relatively high proportion of variation (31%) than that of environmental variables (20%), potentially reflecting spatial patterns linked to species' geographic distributions (Fig. 5). Complete results showing adjusted *r* ^2^ and proportion of variance explained (PVE %) for each model and proportion of variable explained by each environmental variable (Dataset S1).

### Phylogenetic signal and phenotypic variation

**Fig. 6:**
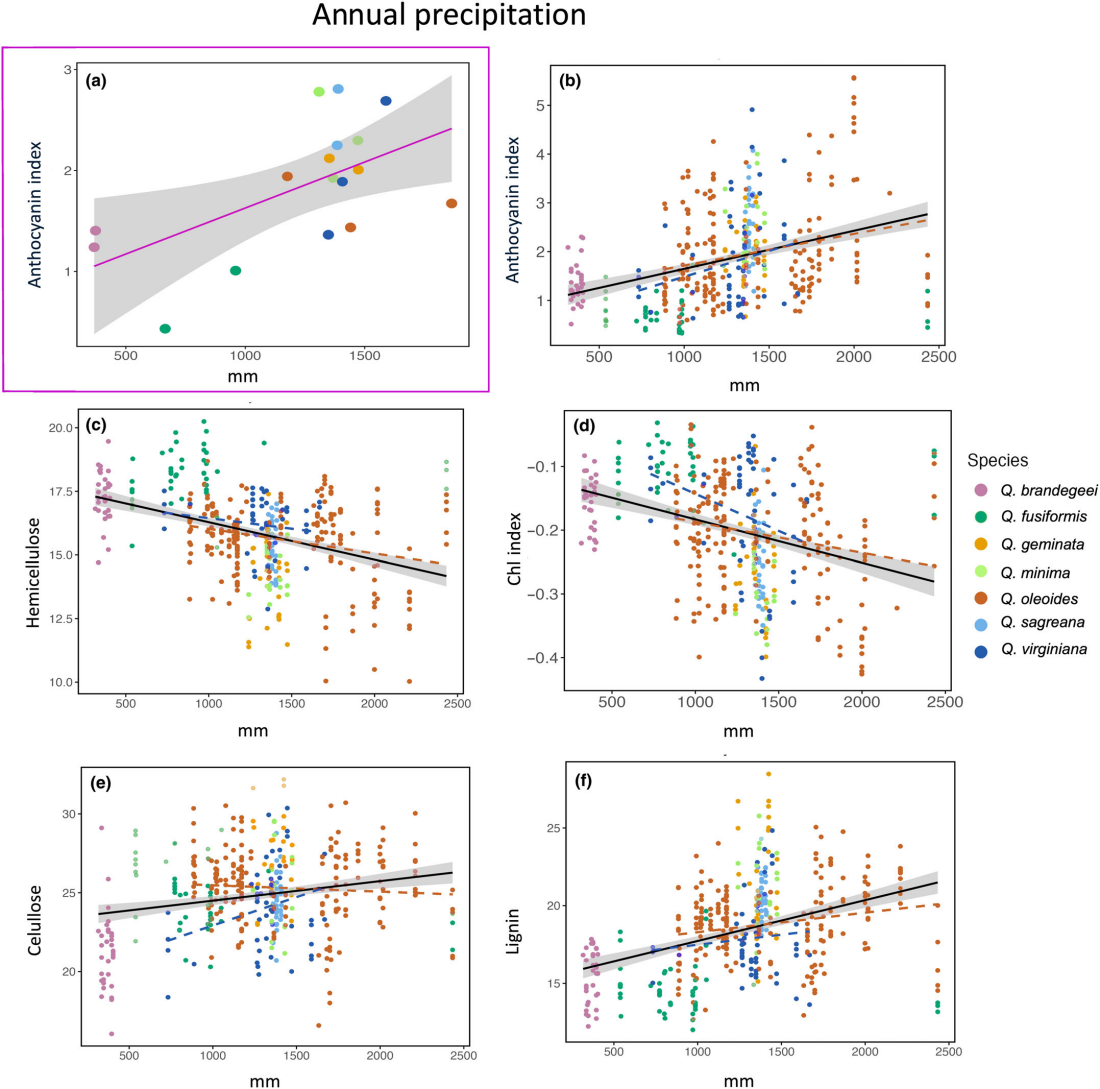
Relationship between traits and annual precipitation. (a) Magenta square‐enclosed graph represents phylogenetic generalized least squares regression (PGLS) model significant association of anthocyanin index and annual precipitation controlled by phylogeny using a phylogenetic tree of 17 individuals. Graphs (b) anthocyanin index, (c) hemicellulose, (d) Chl index, (e) cellulose, and (f) lignin represent significant correlations between annual precipitation and trait measures using 427 individuals. Dashed lines represent significant correlations for individuals of *Quercus virginiana* (blue) and *Quercus oleoides* (vermilion). Shaded areas around regression lines indicate 95% confidence intervals.

**Fig. 7:**
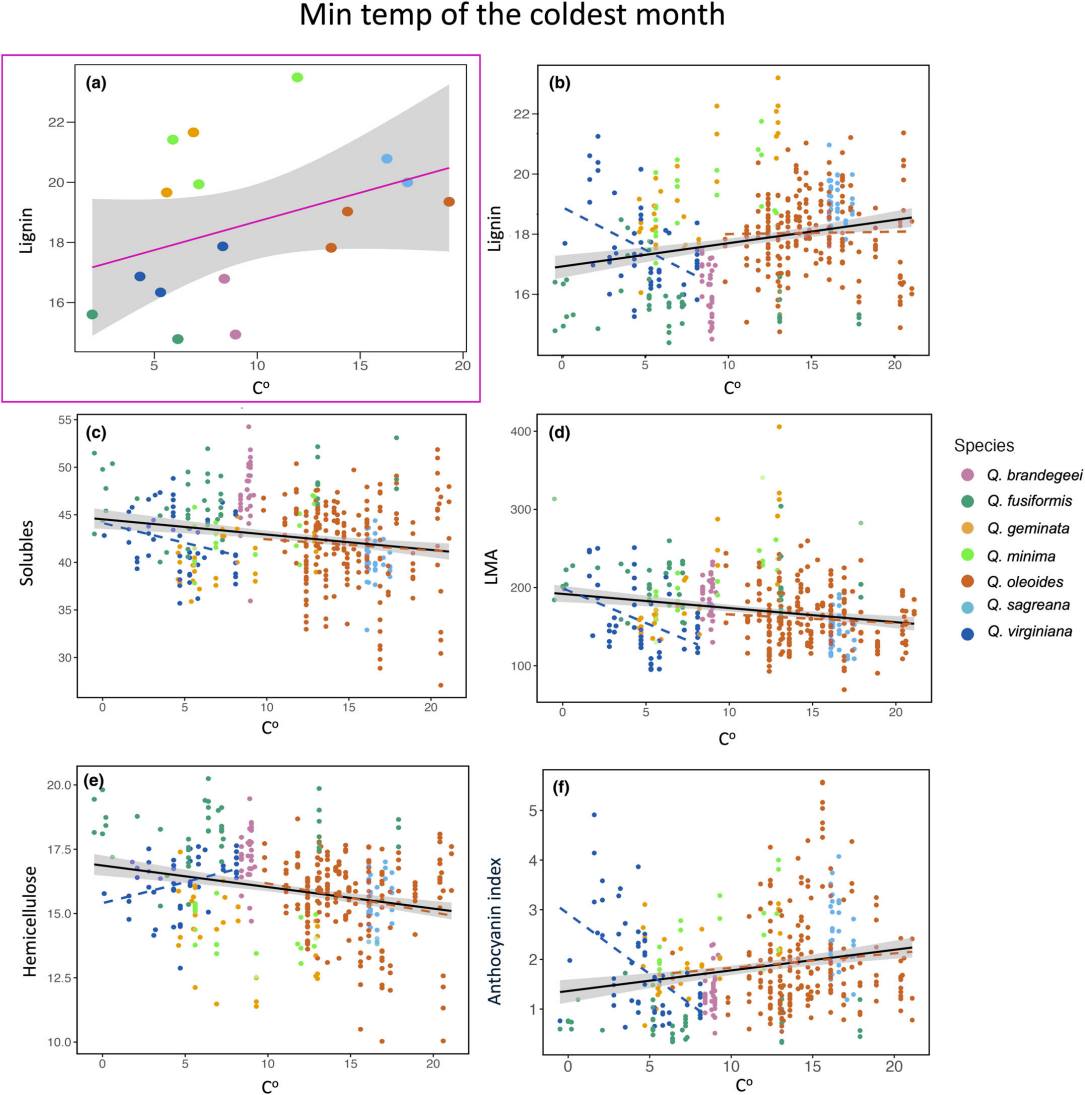
Relationship between traits and minimum temperatures of the coldest month. (a) Magenta square‐enclosed graph represents phylogenetic generalized least squares regression (PGLS) model significant association of Lignin and minimum temperature of the coldest month controlled by phylogeny using a phylogenetic tree of 17 individuals. Graphs (b) lignin, (c) solubles, (d) leaf mass area (LMA), (e) hemicellulose, and (f) anthocyanine index represent significant correlations between minimum temperature of the coldest month and trait measures using 427 individuals. Dashed lines represent significant correlations for individuals of *Quercus virginiana* (blue) and *Quercus oleoides* (vermilion). Shaded areas around regression lines indicate 95% confidence intervals.

Accounting for phylogenetic relationships among populations using PGLS, we found significant associations between traits and several bioclimatic variables. The most relevant were annual precipitation (Bio 12; Fig. 6a) and minimum temperature of the coldest month (Bio 6; Fig. 7a). Additional significant associations were also found with precipitation of the warmest quarter (Bio 18) and mean temperature of the wettest quarter (Bio 8), which are presented in the Table S5 and Fig. S7. Populations that occur in colder regions (e.g. within *Q. fusiformis* and *Q. virginiana*) tended to exhibit lower lignin concentration than those from warmer regions (within *Q. oleoides*, *Q. brandegeei*, and *Q. sagraeana*) (Bio 6), while populations occurring in drier conditions (Bio 12), including those of *Q. brandegeei* and *Q. fusiformis*, showed lower concentrations of anthocyanins as indicated by lower values in the anthocyanin index. Across all 427 individuals, Pearson's correlation coefficients showed significant relationships between traits and the minimum temperature of the coldest month (Fig. 6) for lignin (*r* = 0.24, *P* = 0.0001), cell solubles (*r* = 0.2, *P* = 0.0001), LMA (*r* = 0.21, *P* = 0.0001), hemicellulose (*r* = 0.24, *P* = 0.0001), anthocyanin index (*r* = 0.23, *P* = 0.0001), and for annual precipitation (Fig. 7) for anthocyanin index (*r* = 0.4, *P* = 0.0001), hemicellulose (*r* = 0.4, *P* = 0.0001), Chl index (*r* = 0.4, *P* = 0.0001), cellulose (*r* = 0.21, *P* = 0.0001), and lignin (*r* = 0.4, *P* = 0.0001).

We found significant levels of phylogenetic signals in the spectra of *Quercus* section *Virentes*, with strong signals observed in the visible and some parts of the NIR regions. Predicted traits such as hemicellulose, cell soluble concentrations, and lignin also exhibited strong‐to‐moderate phylogenetic signals (Table S6). By contrast, the SWIR region showed limited phylogenetic signals, suggesting that this region may be less influenced by evolutionary constraints (Fig. S8).

### Accounting for environmental influence on phenotypes using spectral variation in leaves from a common garden

Common garden results for *P*_ST_–*F*_ST_ comparisons in *Q. fusiformis*, *Q. geminata*, *Q. oleoide*s, and *Q. virginiana* generally supported the results for wild populations (shown at a *c* : *h* ^2^ ratio of 0.75 in Fig. S9). Notably, hemicellulose, cellulose, lignin, thickness, and LMA emerged as traits in which *P*_ST_ exceeded *F*_ST_ at least in some pairwise comparisons in both common garden and wild population individuals. Not all pairwise results that were significant in wild populations remained significant in common gardens, yet many were. For example, in both common garden and wild populations, cellulose values for *P*_ST_ exceeded *F*_ST_ for *Q. virginiana* vs *Q. fusiformis* and for *Q. oleoides* vs *Q. fusiformis* but no other pairwise comparisons. For lignin, *P*_ST_ values exceeded *F*_ST_ values for *Q. geminata* vs *Q. virginiana* and *Q. oleoides* vs *Q. fusiformis* in both common garden and wild populations but not in other pairwise comparisons.

## Discussion

Our study highlights the capability of spectral phenotypic data combined with neutral genetic variation to reveal evolutionary processes that have shaped diversification within *Quercus* section *Virentes*. Across the range of the live oak lineage that spans temperate and tropical environments, spectral variation provides key insights into how environmental pressures shape phenotypic traits and drive ecological divergence, complementing genetic analyses for a more comprehensive view of adaptive evolution.

### Sympatric species with niche specialization show evidence for adaptive differentiation

We found that among sympatric *Quercus* species in the southeastern United States (*Q. virginiana*, *Q. geminata*, and *Q. minima*), environmental selection, rather than geographic proximity, drives phenotypic divergence consistent with the hypothesis that fine‐scale adaptations to contrasting local habitats enable coexistence. Our pRDA shows that environmental variables explain more phenotypic variation than geographic distance, even with gene flow. Traits such as LMA, lignin concentrations, cellulose, thickness, and spectral features that differentiate species are likely under selection. These traits and phenotypic attributes may contribute to survival across hydrologically distinct microhabitats. Previous studies have shown linkages between functional trait variation and topography or microhabitat in this region. The topography of northern central Florida forms an ecological gradient in which small elevation changes cause shifts in water availability (Brown, Stone & Carlisle, 1990). *Quercus* species in this area occupy habitats that span xeric sandhills to mesic river edges and ravines (Kurz & Godfrey, 1962; Myers, 1992), resulting in selective pressures that shape functional traits and adaptation to hydrological regimes (Cavender‐Bares *et al*., 2004b; Cavender‐Bares & Holbrook, 2001; Reich *et al*., 2003). Evolutionary divergence among Virentes, *Q. virginiana*, *Q. geminata*, and *Q. minima* has also been found despite complex introgression (Eaton *et al*., 2015; Cavender‐Bares *et al*., 2015). These species maintain reproductive isolation through phenology, niche specialization, and habitat preference (Cavender‐Bares *et al*., 2004a; Cavender‐Bares & Pahlich, 2009). Full spectral analysis reveals strong adaptive divergence among these species, especially in the NIR (700–800 nm) and VIS (616, 688 nm) regions, indicating differences in leaf structure and pigments associated with water availability (Ourcival *et al*., 1999). Although *Q. geminata* and *Q. minima* show little differentiation in the SWIR and do not differ significantly in most of the structural traits (except for cellulose, Fig. 4a), both differ markedly from *Q. virginiana*. Lower lignin concentrations in *Q. virginiana* compared with *Q. geminata* and *Q. minima*, as well as reduced LMA and lower leaf thickness, are trait differences associated with decreased sclerophylly expected in more mesic habitats (Alonso‐Forn *et al*., 2020, 2023; Sancho‐Knapik *et al*., 2021).

In *Q. oleoides*, phenotypic variation across populations also reflects strong adaptive processes. Populations from Mexico show greater freezing tolerance and higher lignin and cellulose concentrations than those in Belize and Costa Rica, adaptations likely shaped by regional climatic differences. Phenotypic clustering in Central American populations is not mirrored by genetic differentiation, suggesting local adaptation and phenotypic plasticity play key roles (Koehler *et al*., 2012; Forester *et al*., 2016; Ramírez‐Valiente & Cavender‐Bares, 2017). The wide geographic range of *Q. oleoides* exposes populations to distinct rainy season dynamics (Deacon & Cavender‐Bares, 2015; Ramírez‐Valiente & Cavender‐Bares, 2017). High genetic diversity, particularly in alleles linked to trade‐offs between drought tolerance and growth rate, provides resilience against extreme climatic events and supports long‐term persistence (Teixeira & Huber, 2021; Meireles *et al*., 2020; Cavender‐Bares, 2019). This flexibility is critical for survival across diverse environmental conditions, reinforcing the role of adaptive processes in shaping phenotypic variation within and among populations of *Q. oleoides*.

### Phenotypic clustering across regions is associated with shared ancestry, historical gene flow, and climatic similarity

Unsupervised clustering analyses (GENELAND) based on spectrally predicted traits place *Q. oleoides* (Central America populations), *Q. sagraeana* (Cuba), and *Q. virginiana*, *Q. geminata*, and *Q. minima* (Florida) into the same cluster (Fig. 2b).

Phenotypic clustering among distinct species can be a consequence of shared ancestry or historical gene flow followed by vicariance. The origin of *Q. sagraeana*, an allopatric Cuban endemic, has been long investigated: one hypothesis suggests migration from Florida (Santiago‐Valentin & Olmstead, 2004; Graham, 2010; Gugger & Cavender‐Bares, 2013), while another supports a Central American origin (Muller, 1955; Eaton *et al*., 2015). Genome‐wide RADseq analyses reveal *Q. sagraeana* as a sister species to *Q. oleoides*, supporting a Central American origin with later introgression from *Q. virginiana* and *Q. geminata* (Eaton *et al*., 2015). Despite restricted gene flow – especially with current high sea levels (Gugger & Cavender‐Bares, 2013) – the lack of clear phenotypic structure may reflect retained ancestral polymorphisms. Phenotypic clustering may also be a consequence of similar selection pressures, given that these populations all occur in subtropical humid environments. In contrast to clustered phenotypes associated with shared ancestry, historical gene flow, and climatic similarity, populations of the allopatric sister species *Q. brandegeei* and *Q. fusiformis* show geographically structured phenotypes that align with genetic differences. *Quercus fusiformis* and *Q. brandegeei* diverged *c*. 5.2 Ma. *Quercus brandegeei*, which occurs in an isolated region of southern Baja California's desert (Cavender‐Bares *et al*., 2015), exhibits traits more suited to aridity, such as smaller and thicker leaves with higher LMA, compared to its sister species. These findings highlight ecological divergence driven by environmental selection between contrasting climates, with pRDA supporting spectral trait differentiation, especially in the NIR region (Fig. 5).

Collectively, the patterns reveal different evolutionary processes: contrasting environmental pressures and microhabitat‐level differentiation drive phenotypic divergence, while shared history and ancestral gene flow, coupled with shared environments, promote phenotypic clustering. Plasticity is also likely to contribute to the patterns of phenotypic variation we observe in wild populations, given the general pattern of greater *P*_ST_–*F*_ST_ differences in wild populations than common garden populations (Fig. S8).

### Phylogenetic history, signal, and environmental influences on phenotypic variation

In *Quercus* section *Virentes*, species distributed across temperate and subtropical climates exhibit significant phenotypic variation influenced by both phylogenetic history and environmental pressures. Using a PGLS model that included several populations per species, we found that species from colder latitudes, such as *Q. fusiformis* and *Q. virginiana*, exhibited lower lignin concentrations in their leaves. This same trend emerges when examining variation at the level of individuals. Individuals across the *Virentes* showed an increase in soluble sugars and hemicellulose in colder climates, consistent with findings in other plant species, including *Arabidopsis* (Panter *et al*., 2019; Kutsuno *et al*., 2023). These chemical changes found in populations from colder climates – lower lignin concentrations coupled with higher cellulose and soluble cell constituents – may be associated with biochemical modifications to the cell wall that enhance freezing tolerance by stabilizing cell structures and facilitating water movement during freeze–thaw cycles (Kutsuno *et al*., 2023). Lower lignin levels are linked to cell permeability, which facilitates water outflow and ice formation in extracellular spaces without damaging cells (Yamada *et al*., 2002; Domon *et al*., 2013; Cass *et al*., 2015). The cell wall plays a crucial role in protecting the plasma membrane from extracellular freezing damage, as it serves as the primary site of ice crystal formation (Panter *et al*., 2020). In *Arabidopsis*, alterations in the pectin cross‐link structure, lignin biosynthesis (Huang *et al*., 2010), and modifications in hemicellulose composition have been shown to affect basal freezing tolerance (Shi & Yang, 2014; Panter *et al*., 2019). Lignins and other phenolic compounds can also act as defense agents affecting leaf optical properties in the SWIR2 region (Li *et al*., 2023).

Previous studies show that the minimum temperature of the coldest month predicts freezing tolerance and cold acclimation capacity in live oaks. In common garden experiments, species from temperate latitudes *Q. virginiana*, *Q. geminata*, and *Q. fusiformis* demonstrated the ability to increase freezing tolerance in response to chilling growth temperatures, contrary to tropical species such as *Q. oleoides* (Koehler *et al*., 2012; Cavender‐Bares, 2007). The ability to cold acclimate and express higher freezing tolerance under temperate conditions was associated with less competitive growth rates under tropical (nonstressed) growth conditions (Koehler *et al*., 2012).

Across individuals, significant variation in ARI is evident within *Q. virginiana* and *Q. oleoides* (Fig. 6b), reflecting their broad geographic and climatic ranges. Within *Q. virginiana*, the highest levels of anthocyanins occur in the coldest climates, but the overall trend across the *Virentes* is one of increasing anthocyanin concentration in warmer, more tropical regions. Accumulation of anthocyanins can contribute to photoprotective effects under cold conditions but can also deter herbivores (Gould, 2004). Under low temperatures, anthocyanins have been shown to mitigate photodamage by intercepting light or neutralizing reactive oxygen species (Pietrini *et al*., 2002; Gould, 2004; Hughes *et al*., 2012). Ramirez‐Valiente *et al*. (2015) reported that anthocyanin levels in immature leaves of *Virentes* species and populations increased in response to seasonal low‐temperature stress, but that across all *Virentes* populations, those from tropical regions exhibited higher anthocyanin levels than those from temperate regions. The latter result points to the role that anthocyanins play in defense against herbivores.

Herbivore pressure is well known to increase at tropical latitudes (e.g. Coley & Barone, 1996; Salazar & Marquis, 2012; Tang *et al*., 2023), and defense chemistry has been shown to increase in more tropical regions in oaks (Pearse & Hipp, 2012). In warmer climates, higher anthocyanin concentrations have been linked to reduced herbivory, including in *Q. robur* (Valdés‐Correcher *et al*., 2025). Our findings of increasing anthocyanin levels in tropical regions indicate that anthocyanins play a greater role in protection against herbivores than in cold tolerance in the *Virentes*. The coupled increase in both anthocyanins and lignin concentrations in tropical regions (Fig. 6d,f) indicates that both may be important elements in defense against herbivores.

We also see a significant trend in increased anthocyanin levels with mean annual precipitation (Fig. 7). *Quercus brandegeei* and *Q. fusiformis*, which experience significant seasonal drought, have lower anthocyanins than species from more mesic environments. Ramirez‐Valiente *et al*. (2015) reported that anthocyanin accumulation was more pronounced in mesic ecotypes under drought conditions, emphasizing that anthocyanins play a key role in mitigating photodamage through their antioxidant and light‐filtering properties in environments with higher water availability because other photoprotective mechanisms involving the xanthophyll cycle are absent. They concluded that mesic populations are more reliant on anthocyanin‐based photoprotection. Interestingly, our results also show that *Q. brandegeei* and *Q. fusiformis* exhibited higher Chl indices despite their lower anthocyanin levels, suggesting that xeric species may prioritize maintaining high photosynthetic efficiency during a shorter active growing season. The observed trade‐off between anthocyanin accumulation and Chl concentration in our study (Fig. 7b,d) suggests an investment in high photosynthesis in shorter active growing seasons in xeric populations (Ramírez‐Valiente & Cavender‐Bares, 2017), on the one hand, and increased anthocyanin‐based photoprotection and/or defense chemistry in more mesic conditions, on the other hand. We also found that individuals from drier sites showed somewhat higher LMA (Fig. 7f). Previous studies have shown that xeric ecotypes in species such as *Fagus sylvatica* tend to exhibit reduced anthocyanin levels while relying on morphological adaptations such as increased LMA and higher trichome density to enhance drought tolerance (Camarero *et al*., 2012; García‐Plazaola & Becerril, 2000). These structural and physiological strategies, combined with higher Chl levels, may support efficient energy use and photosynthetic function in regions prone to drought stress. Our findings highlight the multifunctional role of anthocyanins (Gould, 2004) and the importance of environmental context in shaping leaf morphology, pigment dynamics, and carbon compounds across *Quercus* species.

### Balancing evolutionary constraint and adaptive divergence in spectral traits

The strong phylogenetic signal observed in the visible spectrum (Table S6) supports the hypothesis that spectral traits associated with photosynthesis, such as pigment concentrations, are evolutionarily conserved (Meireles *et al*., 2020). Although closely related species such as *Q. geminata* and *Q. minima* exhibit minimal differences in the visible spectrum, these differences are still ecologically relevant, particularly in specific spectral bands associated with environmental variables. When pairwise comparisons include *Q. virginiana*, these differences become more pronounced, reflecting a dual influence of shared ancestry and local adaptation. This suggests that while the visible spectrum captures phylogenetically conserved traits, it also reveals adaptive shifts driven by local environmental pressures (Liu *et al*., 2015). This interplay highlights the potential for evolutionary plasticity within a conserved spectral framework. The significant environmental contribution to visible spectrum variation – particularly the influence of the topographic wetness index (PVE%; Dataset S1) – underscores the importance of microhabitat‐level adaptive divergence in closely related, sympatric species. Given the sympatry of these species, strong environmental contributions, such as those driven by differences in topographic wetness index levels, to variation in the visible spectrum may reflect niche differentiation, enabling co‐occurrence by reducing competition for water resources. Variation in the visible spectrum may reflect niche differentiation, allowing co‐occurrence by reducing competition. This pattern is consistent with the hypothesis that recent, sympatric divergence is often driven by strong ecological pressures acting on traits with direct functional relevance to the environment (Arnegard *et al*., 2014). Even for traits with high phylogenetic signals, species can fine‐tune or modify their traits within the limits of their evolutionary potential to adapt to contrasting environments.

### Using common gardens to decipher the genetic basis of spectral traits and the role of plasticity

The spatial autocorrelation of environmental variables can lead to geographic and neutral differences between populations correlating with environmental conditions, thereby masking adaptive evolutionary processes (Reznick & Ghalambor, 2001; Prentis *et al*., 2008). Consequently, the use of common gardens is crucial for clarifying the roles of different evolutionary forces in populations, as they allow for the assessment of phenotypic variation under controlled environmental conditions. Our examination of spectral features and spectrally derived traits in common garden experiments compared with wild populations increases our understanding of how trait selection varies depending on interaction with the environment. Sympatric (*Q. geminata* and *Q. virginiana*) or parapatric (*Q. fusiformis* and *Q. oleoides*) species may undergo specialization reinforcing species boundaries and influencing the evolution of adaptive traits. While we did not explicitly test for plasticity in this study, the comparison of *P*_ST_–*F*_ST_ in the same populations grown in a common garden or in the wild reveals that there is a significant component of phenotypic variation attributable to plasticity. However, we found that individuals of sympatric or parapatric species show similar values of *P*_ST_ and significant adaptive divergence in both common gardens and wild populations, indicating the fixation of traits in these populations, perhaps as a consequence of species interactions. This phenomenon was not observed when comparing allopatric or widely distributed species, in which plasticity may be more important for persistence. Plasticity within widely distributed species may confer an advantage in adapting to diverse climates, as seen in populations of *Q. oleoides*. Long‐lived trees that inhabit extensive climatic ranges face the challenge of coping with highly variable climatic conditions and dynamic selective pressures on growth and stress tolerance across different space–temporal scales (Meireles *et al*., 2020).

### Conclusions

Our research demonstrates the efficacy of using leaf‐level spectra and spectrally derived traits to elucidate the roles of adaptive divergence and phenotypic plasticity in the persistence of populations of long‐lived organisms. Understanding adaptive divergence among species is a long‐standing challenge in evolutionary biology due, in part, to the complexity of quantifying phenotypic traits for large sample sizes. Our findings show that IBE, rather than geographic proximity, shapes phenotypic divergence among sympatric *Quercus* species, highlighting the importance of adaptive strategies in sustaining regional coexistence among species that occupy diverse microhabitats. In sympatric species, niche specialization plays a key role in diversification, resulting in trait divergence and speciation and contributing to landscape‐level coexistence among closely related species. While we found evidence for adaptive divergence in live oak species, this divergence occurred alongside substantial gene flow within local populations. This interplay between neutral and selective forces highlights the complexity of adaptation, in which phenotypic differentiation arises together with genetic connectivity. Taken together, these patterns underscore the critical roles of natural selection, genetic variation, and phenotypic plasticity in ensuring the long‐term persistence of these species (Teixeira & Huber, 2021; Cavender‐Bares, 2019). Integration of data that capture a high degree of phenotypic and genetic variation represented within and across divergent lineages is critical to deciphering the influence of genetic and environmental factors and their interactions on phenotypes. The approach we used integrates disparate fields within evolutionary biology and ecology to elucidate how interactions between landscapes and species biological traits drive biodiversity patterns.

## Competing interests

None declared.

## Author contributions

All authors contributed intellectually. JC‐B conceived and designed the study, collected the specimens and managed the laboratory. MSH‐L designed the data analysis, conducted genetic and phenotypic analyses, contributed to common garden leaf scanning, designed the figures and wrote the manuscript. JAGQ. developed the spectral trait prediction models and contributed to figure editing. AGR contributed to phenotypic analysis. MSH‐L and JC‐B wrote the manuscript, and all authors edited it.

## Disclaimer

The New Phytologist Foundation remains neutral with regard to jurisdictional claims in maps and in any institutional affiliations.

## Supporting information


**Dataset S1** Results of pRDA analysis, including variance partitioning and environmental variable contributions to phenotypic traits.

**Dataset S2** Results of *P*_ST_‐*F*_ST_ comparisons for predicted traits, VIP wavelength in visible, near‐infrared (NIR), and short‐wave infrared (SWIR) for each pairwise species in the section Virentes for wild populations and *P*_ST_‐*F*_ST_ for predicted traits in glasshouse.

**Fig. S1** Distribution of the seven species in section *Virentes* based on species occurrences and continuous wavelet transform of leaf reflectance spectra.
**Fig. S2** Internal validation results for leaf mass per area, thickness, cell solubles, hemicellulose, cellulose, and lignin predicted from dry spectra.
**Fig. S3** Variable importance in the projection metric was calculated based on dry sample spectral data models for six leaf predicted traits.
**Fig. S4** Spatial distribution of genetic and phenotypic variation in *Quercus oleoides*.
**Fig. S5** *P*_ST_ comparisons among *Quercus oleoides* genetic groups predicted using 11 nuclear simple sequence repeat genetic markers.
**Fig. S6** *P*_ST_ comparisons among species using spectrally predicted traits (leaf mass per area; thickness; cell solubles; hemicellulose; cellulose; lignin); spectral bands within the visible, near‐infrared, and short‐wave infrared region with high importance (i.e. variable importance of projection in discriminating species using wavelet spectra).
**Fig. S7** Spectrally predicted traits and selected wavelength relationships under different environmental conditions.
**Fig. S8** *P*_ST_ vs *F*_ST_ estimates and 95% confidence intervals from quantitative predicted traits among wild and glasshouse individuals for four species in *Quercus* section *Virentes*.
**Fig. S9** Phylogenetic signal detected in leaf spectra varies across wavelengths across *Quercus Virentes* species.
**Methods S1** Environmental characteristics of the *Virentes* lineage.
**Methods S2** Bayesian clustering using the structure software.
**Methods S3** Continuous wavelet transform.
**Methods S4** Partial least squares regression modeling framework to predict leaf traits from dried‐leaf reflectance spectra.
**Methods S5** Comparing phenotypic vs genotypic divergence.
**Methods S6** Spatial and environmental drivers of population divergence.
**Table S1** Previous studies from which the genetic data were obtained and from which specimens were collected and measured for spectral data.
**Table S2** Results of hierarchical analyses of molecular variance for seven species of the *Virentes* section in 64 populations based on 11 nuclear simple sequence repeat (nSSRs) loci; pairwise *F*_ST_ values of genetic differentiation in nSSR among the seven species of *Virentes*; pairwise *F*_ST_ values of genetic differentiation in nSSR among genetic groups of *Quercus oleoides*.
**Table S3** Summary statistics and SD of partial least squares regression discriminant analysis model from pressed‐leaf spectra restricted to 1400–2400 nm.
**Table S4** Summary statistics for the partial least squares regression calibration and validation models for each leaf trait.
**Table S5** Statistical associations between various plant traits, environmental variables, and spectral reflectance at specific wavelengths in three regions visible, near‐infrared, short‐wave infrared, and Spectral INDEX.
**Table S6** Phylogenetic signal calculated using Blomberg's K (K), *K*_WN_ white noise, and *K*_BM_ Brownian motion estimated for the seven species in *Quercus Virentes* phylogeny, where regions with significant signal.
Please note: Wiley is not responsible for the content or functionality of any Supporting Information supplied by the authors. Any queries (other than missing material) should be directed to the *New Phytologist* Central Office.

## Data Availability

All data and code used in this study are publicly available via the Harvard Dataverse: doi: 10.7910/dvn/ea2own. This repository contains: Raw and processed reflectance spectra from dried leaves of *Quercus* section *Virentes* individuals, sampled from both wild populations and common garden experiments. Metadata linking individuals to species, population, and geographic location. Microsatellite genotyping data (nuclear simple sequence repeat). Results from partial redundancy analyses (pRDA) and phenotypic differentiation analyses (*P*_ST_). R scripts used for spectral trait modeling and classification. All files are provided in open formats (.csv, .xlsx, .sig) and include documentation to facilitate reuse. Additional processed datasets and figures are provided in the Supporting Information. No sequence‐based accession numbers are associated with this study.
