# Supplementary material for: Foliar spectral signatures reveal adaptive divergence in live oaks (Quercus section Virentes) across species and environmental niches
Source: New Phytol. 2025 Sep 3;248(1):370–88. doi: 10.1111/nph.70424 (PMC12409106; doi:10.1111/nph.70424)
Supplement: Supplementary file 3 — Fig. S1 Distribution of the seven species in section Virentes based on species occurrences and continuous wavelet transform of leaf reflectance spectra. Fig. S2 Internal validation results for leaf mass per area, thickness, cell solubles, hemicellulose, cellulose, and lignin predicted from dry spectra. Fig. S3 Variable importance in the projection metric was calculated based on dry sample spectral data models for six leaf predicted traits. Fig. S4 Spatial distribution of genetic and phenotypic variation in Quercus oleoides. Fig. S5 P ST comparisons among Quercus oleoides genetic groups predicted using 11 nuclear simple sequence repeat genetic markers. Fig. S6 P ST comparisons among species using spectrally predicted traits (leaf mass per area; thickness; cell solubles; hemicellulose; cellulose; lignin); spectral bands within the visible, near‐infrared, and short‐wave infrared region with high importance (i.e. variable importance of projection in discriminating species using wavelet spectra). Fig. S7 Spectrally predicted traits and selected wavelength relationships under different environmental conditions. Fig. S8 P ST vs F ST estimates and 95% confidence intervals from quantitative predicted traits among wild and glasshouse individuals for four species in Quercus section Virentes. Fig. S9 Phylogenetic signal detected in leaf spectra varies across wavelengths across Quercus Virentes species. Methods S1 Environmental characteristics of the Virentes lineage. Methods S2 Bayesian clustering using the structure software. Methods S3 Continuous wavelet transform. Methods S4 Partial least squares regression modeling framework to predict leaf traits from dried‐leaf reflectance spectra. Methods S5 Comparing phenotypic vs genotypic divergence. Methods S6 Spatial and environmental drivers of population divergence. Table S1 Previous studies from which the genetic data were obtained and from which specimens were collected and measured for spectral data. Table S2 Results of hierarc [file NPH-248-370-s003.docx]

**New Phytologist Supporting Information**
**Article title:** Foliar spectral signatures reveal adaptive divergence in live oaks (*Quercus* section *Virentes)* across species and environmental niches

**Authors:** Mariana S. Hernández-Leal^1*^, J. Antonio Guzmán Q.^1^, Antonio González Rodríguez^2^, Jeannine Cavender-Bares^1*^

**Article acceptance date:** 2 July 2025

**
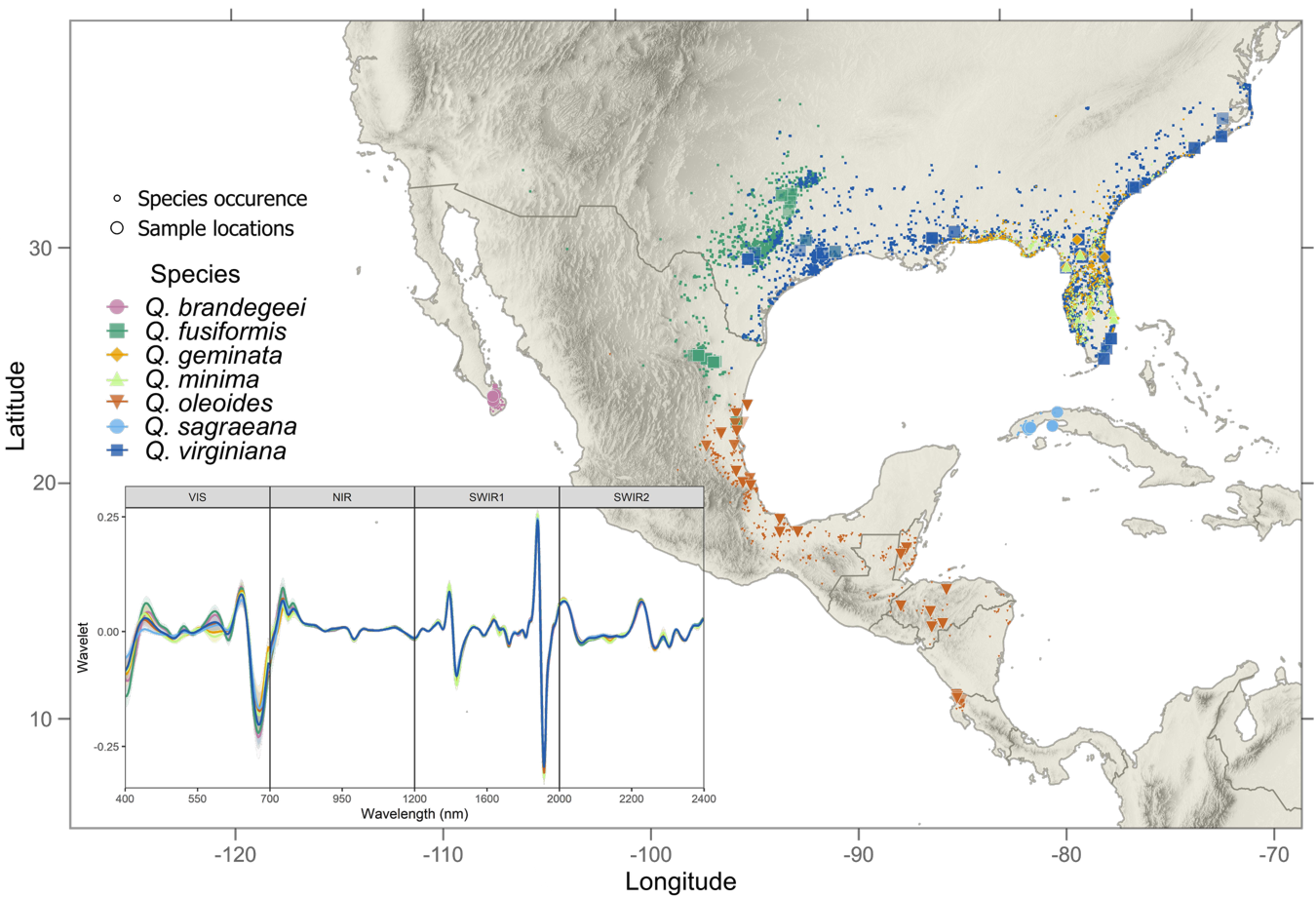
**

**Fig. S1.** **Distribution of the seven species in section Virentes based on species occurrences and Continuous Wavelet Transform (CWT) of leaf reflectance spectra**. Larger points indicate sampled populations used for spectral analysis, while smaller points represent the broader species distribution based on occurrence records. The panel shows the CWT mean spectra and 95% quantiles for each species across the visible (VIS), near-infrared (NIR), and the first and second short-wave infrared regions (SWIR1 and SWIR2).

**Supporting Methods**

**Table S1 Previous studies from which the genetic data were obtained and from which specimens were collected and measured for spectral data.** Studies from which three individual leaves were taken to measure the dry spectrum. Leaves were collected from wild populations and common gardens for DNA extraction, and ecophysiology experiments. A portion of the leaves were pressure dried and stored in a dry cabinet at the University of Minnesota.

|  |  | Species | Data | Comments | Study |
| --- | --- | --- | --- | --- | --- |
|  |  | *Q. geminata, Q. virginiana* | nSSRs |  | Cavender-Bares, & Pahlich,2009 |
|  |  | *Q. geminata, Q. virginiana, Q. oleoides, Q. fusiformis* | Dried leaves for spectra measurments | Only the leaves of individuals on warm treatment were measured | Koehler *et al.* 2011 |
|  |  | *Q. oleoides, Q. virginiana* | nSSRs |  | Cavender- Bares *et al.* 2011 |
|  |  | *Q. sagreana* | nSSRs |  | Gugger & Cavender-Bares, 2013 |

**Methods S1: *Environmental Characteristics of the Virentes Lineage***

The *Virentes* lineage is distinguished within the genus *Quercus* by its restriction to low-altitude habitats, generally on well-drained sandy or volcanic tuff soils (Muller 1961a; Boucher 1983; Nixon 1985; Cavender-Bares et al. 2004a). The species in this lineage share key morphological synapomorphies, such as fused cotyledons and fused stellate trichomes (Candolle 1862; Engelmann 1876-1877; Lewis 1911; Coker 1912; Camus 1936-1938). All Virentes species are wind-pollinated and inter-fertile, with exceptionally high wood density (Nixon 1985; Nixon & Muller 1997) They maintain green foliage throughout winter in the southeastern U.S. and Texas or during the dry season in Central America, with a leaf lifespan of approximately one year. Species within this lineage exhibit varying degrees of tolerance to freezing and drought, which influences their distribution and migration patterns (Cavender-Bares 2007; Cavender-Bares & Pahlich 2009; Koehler et al. 2012).

1. *Quercus virginiana*

*Quercus virginiana* is a large, long-lived tree that grows in a variety of soil types, from moist to well-drained soils, and it can tolerate both alkaline and salty soils. Unlike *Q. minima* and *Q. geminata, Q. virginiana* is less drought-tolerant (Cavender-Bares et al. 2004). Some populations of *Q. virginiana* have shown the ability to tolerate short-term freezing temperatures, withstanding temperatures as low as -18°C to -12°C, making it more cold-tolerant than tropical oaks like *Q. oleoides* but less so than temperate oaks.(Cavender-Bares & Pahlich 2009; Koehler et al. 2012, Fontes et al in preparation).This cold tolerance has allowed it to establish in areas with mild winters.

1. *Quercus geminata*

*Quercus geminata* is a smaller tree relative to *Q. virginiana*, with pubescent leaves that reduce water loss, making it well-suited to arid conditions. It has a robust root system that provides greater stability in sandy soils. The tree prefers sandy, well-drained soils typical of coastal dunes and deep sandy areas. Compared to *Q. virginiana*, it is more tolerant of salt and drought. It can also produce resprouts from its roots, enabling it to form clonal colonies. This combination of traits gives *Q. geminata* a competitive advantage in sandy coastal areas exposed to strong winds and occasional drought.

1. *Quercus minima*

*Quercus minima* is a low-growing shrub that rarely exceeds 2 meters in height. It reproduces both by seed and through underground rhizomes, allowing it to form dense clonal colonies. This species is found in the coastal plains of the southeastern United States, particularly in Florida. It prefers well-drained soils and thrives in thickets and prairies that experience frequent fires. Due to its reliance on rhizome regeneration, Q. minima is more fire-resistant than *Q. geminata* and *Q. virginiana*, as its underground shoots allow for rapid post-fire recovery.

*4. Quercus brandegeei*

*Quercus brandegee*i is an endemic and endangered oak species restricted to the mountainous region of the Sierra La Laguna in Baja California Sur, Mexico. Unlike other oaks, this tree is limited to growth in ephemeral riverbeds, which are seasonally flooded by hurricane waters, making water availability a crucial factor for its survival. (Denvir and Westwood, 2016; Cavender-Bares et al., 2015) *Q. brandegeei* exhibits a clustered spatial distribution, concentrating near these water sources. It relies heavily on riparian environments, where fluctuations in moisture are essential for its survival. The conservation status of *Q. brandegeei* is of significant concern due to its limited range and dependence on specific water availability (Carrero et al.2020).

*5. Quercus fusiformis*

*Quercus fusiformis*, also known as Texas live oak, is native to the southern United States and parts of northern Mexico (Muller, 1961). This species is adapted to dry, mountainous terrain and can be found as far west as Arizona. It is highly tolerant of heat and drought, thriving in arid environments where other oaks struggle. It exhibits frost tolerance to temperatures as low as -12°C , with its survival in colder regions depending on local microclimates and the age or health of the tree (Ramirez-Valiente et al. 2015). Unlike most other species, its evergreen nature allows it to retain leaves year-round, which helps shield the bark and inner tissues from freezing temperatures.

6. *Quercus oleoides*

*Quercus oleoides*, also known as the tropical live oak, is a key species in the seasonally dry tropical forests of Central America, with its range extending from northern Mexico through Costa Rica. It prefers warm, dry climates with limited exposure to freezing temperatures. This species thrives in nutrient-poor, sandy soils or volcanic tuff and forms part of the monodominant stands of tropical dry forests (Cavender-Bares, 2005). Unlike temperate oak species, *Q. oleoides* is not highly tolerant to cold. It can endure brief, mild cold spells, but prolonged frost or subfreezing temperatures are lethal to the species. This thermal limitation reflects its adaptation to tropical and subtropical conditions (Cavender-Bares et al. 2011).

7. *Quercus sagraeana*

*Quercus sagraeana*, also known as the Cuban oak, is a medium-sized evergreen tree that is endemic to western Cuba. It is the only oak species native to the Caribbean. This species thrives in the seasonally dry tropical biome, where it is adapted to warm, dry conditions with pronounced wet and dry seasons. Its habitat includes the Cuban pine forests ecoregion, and its survival is linked to its ability to endure the seasonal variability of water availability *(*Gugger & Cavender-Bares 2013).

**Method S2 Bayesian clustering using STRUCTURE software.**

Bayesian analysis was used both to define the genetic groups among the seven *Virentes* species and, separately, to identify population-level genetic structure within the broadly distributed *Quercus oleoides*. For the interspecific analysis, we included all 427 individuals spanning the geographic range of the seven species. For the within-species analysis, we selected 201 individuals of *Q. oleoides* representing its entire latitudinal and environmental distribution from northern Mexico to Costa Rica.

To quantify genetic differentiation, we conducted an analysis of molecular variance (AMOVA) comparing species and genetic groups using ARLEQUIN v3.5.1.2 (Excoffier & Lischer, 2010) under the infinite alleles model (*F*_ST_). In addition, we calculated pairwise genetic differentiation between species and among STRUCTURE-defined genetic groups using *F*_ST_ estimators implemented in the **hierfstat** package in R (Goudet, 2005).

**Method S3 Continuous Wavelet Transform**

The Continuous Wavelet Transform (CWT) is a method used to analyze localized variations of power within a signal at multiple scales, enabling the decomposition of a signal into its frequency components (Graps 1995). Unlike Fourier transforms, which analyze signals in a fixed global frequency domain, the CWT provides a multi-scale representation, making it particularly suited for analyzing complex, non-stationary signals such as reflectance spectra.

In our study, we applied the CWT to [briefly describe what the spectra or data represent, e.g., leaf reflectance spectra] to identify spectral patterns associated with [specific traits or properties]. The CWT transforms the original signal into a set of wavelet coefficients that correspond to different scales (or frequencies). Each scale reflects patterns or features at a specific resolution, with smaller scales capturing finer details and larger scales representing broader trends.

Summing Wavelet Scales:

To summarize information across relevant scales, we summed the wavelet coefficients within predefined ranges of scales. These ranges were chosen based on [criteria, e.g., prior studies, known spectral regions related to traits]. Summing across scales provides an aggregate metric that captures the overall contribution of specific spectral features to the traits of interest. This approach is analogous to integrating over a frequency band but tailored to the multi-scale nature of wavelet analysis.

**Method S4 PLSR modeling framework to predict leaf traits from dried-leaf reflectance spectra.**

For each sample, we measured full-range reflectance spectra (350–2,500 nm) of the leaves. To construct the models, we measured the following leaf structural and chemical traits on a percentage of the samples of *Quercus Virentes* species plus other *Quercus* species: LMA (kg/m2), thickness (mm), carbon fractions (soluble cell contents, hemicellulose, cellulose, and lignin; %), and concentrations of a variety of elements (Al, C, Ca, Cu, Fe, K, Mg, Mn, N, Na, P, Zn; % or mg/g). We used a PLSR modelling framework to predict each trait from pressed-leaf spectra across the full range (400–2,400 nm). PLSR is suited to handle spectral datasets, which have many collinear predictors, because it projects the spectral matrix onto a smaller number of orthogonal latent components in a way that maximizes the ability to predict the response variable. Our methods for model calibration and validation largely follow Burnett *et al.* (2021). To avoid the imbalance given by the number of samples of each species, the data were split hierarchically using the species as an index to maintain a proportion close to 60% samples for calibration and 40% for independent validation datasets (Appendix Table 1 list the number of individuals used for each trait prediction, the smallest number of components selected). We quantified model performance using R2 and root mean squared error (RMSE) between measurements and mean predictions. We also report the RMSE as a percentage of the 2.5% trimmed range of measured values (%RMSE), which we used rather than the entire range (as in e.g. Burnett *et al.*, 2021) for robustness to outliers. For each trait, we also tested whether the magnitude of residuals (observed minus predicted) in the validation dataset varied among leaves with different discoloration scores. We performed all statistical analyses in R v. 3.6.3 (R Core Team, 2020) and used package pls v. 2.7.1 (Mevik *et al.*, 2019) for PLSR modelling.

**Method S5 Comparing phenotypic vs genotypic divergence.**

The *Q*_ST_ index introduced by Lande (1992) and Spitze (1993) is intended as an analog to *F*_ST_ by measuring the degree of phenotypic variance among populations over a set of quantitative traits rather than at a specific locus. *F*_ST_ can be used as a null hypothesis by assuming that the value of *F*_ST_ measured through neutral loci is the value of divergence between populations due to drift and migration. Assuming neutrality (no selection), we thus expect that *F*_ST_ = *Q*_ST_, meaning that divergence between traits (or phenotypic characters) could be achieved by drift alone. If *Q*_ST_ > *F*_ST_ the inference is that quantitative traits show a higher level of differentiation than expected by genetic drift, assuming directional selection by favoring different phenotypes (i.e., heterogeneous selection). If *Q*_ST_ < *F*_ST_ trait divergence among populations is less than expected by drift alone, indicating the influence of natural selection, but one that is selecting for the same optimum in different populations (i.e., stabilizing selection).

In wild populations where imposing a breeding design is challenging, the *Q*_ST_ index is often approximated by *P*_ST_ (Leinonen et al., 2006). The difference between *Q*_ST_ and *P*_ST_ is that the latter is calculated from phenotypic variance components with no distinction between the relative contribution of genetic and environmental variation:

$P_{ST}=\frac{{\frac{c}{h^{2}} \sigma}_{b}^{2}}{\frac{c}{h^{2}} \sigma_{b}^{2}+2\sigma_{w}^{2}}$ _(eqn. 1)_

where $\sigma_{b}^{2}$ and $\sigma_{w}^{2}$ are the phenotypic variance components between and within populations, *h*^2^ is heritability (i.e., the proportion of phenotypic variance due to additive genetic effects), and *c* is an estimate of the proportion of the total variance due to additive genetic effects across populations (Brommer, 2011).

Given that traits were measured in wild populations rather than in common garden experiments designed to estimate heritability (*h^2^*) and additive genetic variation (c), and that the *P*_ST_ approximation of *Q*_ST_ is dependent on how well *c* and *h* are theoretically calculated, we estimated the *c*/*h*^2^  ratio following Seeholzer & Brumfield (2018):

$\frac{c}{h^{2}}=\frac{{{-2F}_{ST(upper)}\sigma_{W(upper)}^{2}}}{\sigma_{B(lower)}^{2}+{(F}_{ST(upper)}-1)}$ _(eqn 2)_

Where  $\sigma_{W(upper)}^{2}$ is the upper confidence interval (CI) value for the within-populations phenotypic variance, $\sigma_{B(lower)}^{2}$ is the lower CI value for the between populations phenotypic variance and *F*_ST_ (upper) is the upper CI value estimated from the genetic markers (*F*_ST_). Ratios of *c*/*h²* closer to zero are considered robust evidence that *P*_ST_ (phenotypic differentiation among populations) surpasses *F*_ST_ (genetic differentiation among populations), indicating a deviation from neutral expectations (Brommer, 2011). Following this rationale, we interpret *c*/*h²* ratios less than 0.25 as strong evidence for natural selection driving phenotypic differentiation, ratios of 0.26–0.50 as moderate selection, and values between 0.51–0.75 as weak selection. We interpret values approaching or exceeding one as very weak or no selection. In the latter case, differentiation results primarily from random changes (i.e., genetic drift), plasticity, and environmental effects (Brommer, 2011, Cruz-Nicolas *et al.* 2019). Several studies have detected that the leaf weight-area ratio exhibits allometric growth; (Niklas *et al.* 2007, 2009; Li *et al.* 2008; Niklas & Cobb 2008, Sun *et al.* 2017; Lin *et al.* 2018). Since both the traits predicted from the spectrum and the wave-lengths are affected by leaf area and density, all phenotypic traits were transformed using the Aitchison log-ratio transformation (Aitchison, 1986).


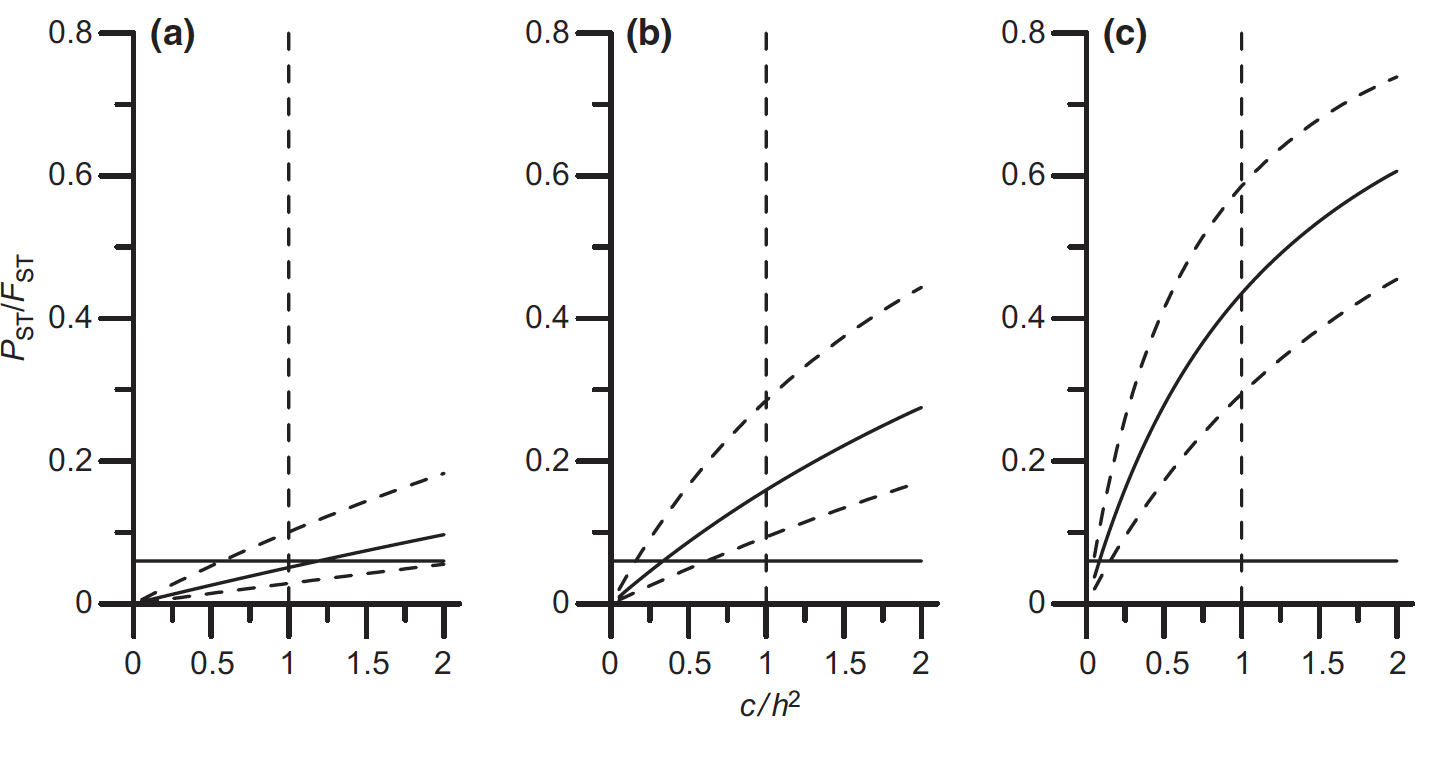


**Fig.** Plots illustrating how a comparison between neutral differentiation and the estimate of *P*ST [sensu eqn (2)] depends on the c ⁄ h2 ratio. In each plot, the dashed vertical line indicates the point of the ‘null assumption’ c = h2 for estimating *P*ST. The horizontal line marks the upper confidence estimate of the neutral divergence estimated as *F*ST (= 0.06 in each plot). Estimates of *P*ST and its lower and upper 95% confidence intervals are plotted. Panel (a) indicates low *P*ST (95% CI at c = h2: 0.029–0.107), where PST clearly does not differ from *F*ST. Panel (b) is for a trait where *P*ST > *F*ST for the null assumption (95% CI: 0.0938–0.285), but the significance of this difference is not very robustas the lower confidence estimate of *P*ST overlaps with the upper confidence estimate for *F*ST when c ⁄ h2 = 0.63. Panel (c) indicates a trait with strong phenotypic divergence (*P*ST 95% CI at c = h2: 0.2946–0.586), and the difference in *P*ST and *F*ST is robust as their confidence intervals only overlap when c ⁄ h2 = 0.17.(Taken from Brommer 2011).

**Method S6 *Spatial and environmental drivers of population divergence****.*

To quantify environmental variation across distribution of the seven species of Virentes we used the 19 Environmental variables extracted from the WorldClim database and the HYDRO1k (USGS, 2024) at 30s arc resolution. Hydro 1k raster data sets are the hydrologically correct DEM, derived flow directions, flow accumulations, slope, aspect, and a compound topographic (wetness) index. Before any analyses, the pool of environmental variables was reduced from 24 to 6 after removing highly correlated variables (based on Pearson's correlation |r| ≤ 0.60) to control for multicollinearity for each pairwise species. We chose to perform pairwise comparisons involving Q.

*oleoides* by selectively including only populations that showed genetic or phenotypic concordance with the other species under comparison. This decision was driven by the broad geographic distribution and high intraspecific variability of *Q. oleoides*, which could obscure meaningful interspecific patterns if all populations were included indiscriminately. We chose to perform pairwise comparisons involving *Q. oleoides* by selectively including only those populations that exhibited genetic or phenotypic concordance with the other species being compared. This decision was based on the broad geographic range and high intraspecific variability of *Q. oleoides*, which could mask meaningful interspecific patterns if all populations were included without distinction. For instance, only the Northern Mexico populations of *Q. oleoides* that show evidence of admixture with *Q. fusiformis* were used in comparisons with that species. Similarly, only Central American populations of *Q. oleoides* were included in pairwise pRDA analyses with the Florida and Cuban populations of *Q. virginiana*, *Q. sagreana*, *Q. minima*, and *Q. geminata*. Environmental variables used in the analysis captured variation in temperature, precipitation, and soil water capacity. Geographic context was represented by population latitude and longitude. Genetic distance was calculated among all 427 individuals using pairwise Nei’s D (Nei, 1972) in GENALEX. These distances were summarized via Principal Coordinates Analysis (PCoA), and the first five PCoA axes were used as composite variables to represent genetic structure.

We quantified the degree to which phenotypic variation was assessed using a variance partitioning technique to estimate individual and shared contributions of each variable (Borcard et al., 1992). This approach uses partial RDAs to estimate the proportion of explained variance for each predictor variable, independently and combined, out of the total explained variance. Each model included phenotype as the response variable and all combinations of genetic, environmental, and geographic variation as predictors. Phenotype was characterized by four variables: the six traits predicted from the PLSR spectral modelling, and the VIP wavelength bands that had *P*_ST_ > *F*_ST_ significant values for each pairwise species. The total phenotypic variance explained (PVE) by the predictors and adjusted r^2^(i.e., individual contribution in terms of total PVE) was estimated with the varpart function in the vegan package in R. Model significance, when appropriate, was assessed using independent RDA and pRDAs, permutation based. ANOVAs (n = 999), and significance thresholds of α ≥ 0.05. All measurements were centered and standardized prior to analyses.

**Table S2. a) Results of hierarchical analyses of molecular variance (AMOVA) for 7 species of the *Virentes* section in 64 populations based on eleven nSSRs loci. b) Pairwise *F*_ST_ s of genetic differentiation in nSSR among the seven species of *Virentes* c)** **Pairwise *F*_ST_ values of genetic differentiation in nSSR among genetic groups of *Q. oleoides*.** Values in bold numbers are significant *p* < 0.05. GE= *Q. geminate*, MN= *Q. minima*, VI= *Q. virginiana*, SA= *Q. sagraeana*, OL= *Q. oleoides*, BR= *Q. brandegee*i, FU= *Q. fusiformis.*

**a)**

| Level | df | SS | VC | % |  |
| --- | --- | --- | --- | --- | --- |
| Among species | 6.0 | 500.1 | 0.4 | 9.2** |  |
| Among populations within species | 57.0 | 627.3 | 0.3 | 6.5** |  |
| Among individuals within populations | 605.0 | 2792.6 | 0.6 | 13.7** |  |
| Within individuals | 669.0 | 2226.5 | 3.3 | 70.6** |  |
| Total | 1337.0 | 6146.5 | 4.7 |  |  |

df, degrees of freedom; SS, Sum of Squares; VC, Variance Components; *p <0.05, **p < 0.001

**b)**

|  | GE | MN | VI | OL | SA | BR | FU |
| --- | --- | --- | --- | --- | --- | --- | --- |
| GE |  | **0.012** | **0.130** | **0.162** | **0.233** | **0.272** | **0.175** |
| MN |  |  | **0.111** | **0.138** | **0.214** | **0.244** | **0.146** |
| VI |  |  |  | **0.059** | **0.100** | **0.167** | **0.079** |
| OL |  |  |  |  | **0.073** | **0.185** | **0.046** |
| SA |  |  |  |  |  | **0.243** | **0.146** |
| BR |  |  |  |  |  |  | **0.152** |
| FU |  |  |  |  |  |  |  |

| **c)** |  |  |  |  |  |  |  |
| --- | --- | --- | --- | --- | --- | --- | --- |
|  | **K1** | **K2** | **K3** | **K4** |  |  |  |
| **K1** |  | **0.04** | **0.076** | **0.132** |  |  |  |
| **K2** |  |  | **0.028** | **0.109** |  |  |  |
| **K3** |  |  |  | **0.058** |  |  |  |
| **K4** |  |  |  |  |  |  |  |

****p* <0.05**

**Table S3.** Summary statistics and SD of PLSR_DA model from pressed-leaf spectra restricted to 1400-2400 nm. Using Raw spectral data, vector Normalized and Continuous Wavelet Transform.

|  | Raw | Vector Normalized | CWT |  |
| --- | --- | --- | --- | --- |
| Accuracy | 0.92 ± 0.011 | 0.91 ± 0.012 | 0.92 ± 0.011 |  |
| Kappa | 0.88 ± 0.018 | 0.86 ± 0.019 | 0.88 ± 0.017 |  |
| Sensitivity | 0.88 ± 0.10 | 0.87 ± 0.10 | 0.87 ± 0.10 |  |
| Specificity | 0.98 ± 0.016 | 0.98 ± 0.017 | 0.89 ± 0.014 |  |

**Table S4. Summary statistics for the PLSR calibration and validation models for each leaf trait**. #S: number of samples, #C: number of components. Performance statistics include R^2^: the fit between the observed values and the predicted values, RMSE: root mean square error, %RMSE: percent root mean square error, MAE: mean absolute error. spectrally predicted traits (LMA: Leaf mass area; THI: thickness; SOL: solubles; HEM: hemicellulose; CEL: cellulose; LIG: lignin).

|  |  |  |  | **Calibration model** | | | | | **Validation model** | | | |
| --- | --- | --- | --- | --- | --- | --- | --- | --- | --- | --- | --- | --- |
| **Trait** | **# S** | **# C** | **data**  **range** | **^R2^** | **RMSE** | **%RMSE** | | **MAE** | **^R2^** | **RMSE** | **%RMSE** | **MAE** |
| **^LMA g/m-2^** | 146 | 6 | 33.8 – 273.6 | 0.87 | 19.21 | 0.15 | 14.51 | | 0.88 | 17.6 | 0.14 | 13.25 |
| **THI mm** | 151 | 6 | 0.101 – 0.75 | 0.82 | 0.06 | 0.2 | 0.04 | | 0.65 | 0.08 | 0.28 | 0.06 |
| **SOL %** | 84 | 8 | 36.5 – 61.9 | 0.86 | 2.4 | 0.05 | 1.87 | | 0.51 | 4.29 | 0.09 | 3.63 |
| **HEM %** | 74 | 8 | 11.3 – 0.9 | 0.79 | 1.06 | 0.06 | 0.83 | | 0.25 | 2.07 | 0.12 | 1.61 |
| **CEL %** | 97 | 9 | 8.6 – 32.6 | 0.84 | 2.28 | 0.11 | 1.78 | | 0.72 | 3.37 | 0.17 | 2.64 |
| **LIG %** | 96 | 8 | 7.1 – 23.8 | 0.74 | 2.72 | 0.17 | 0.14 | | 0.59 | 2.72 | 0.17 | 0.17 |


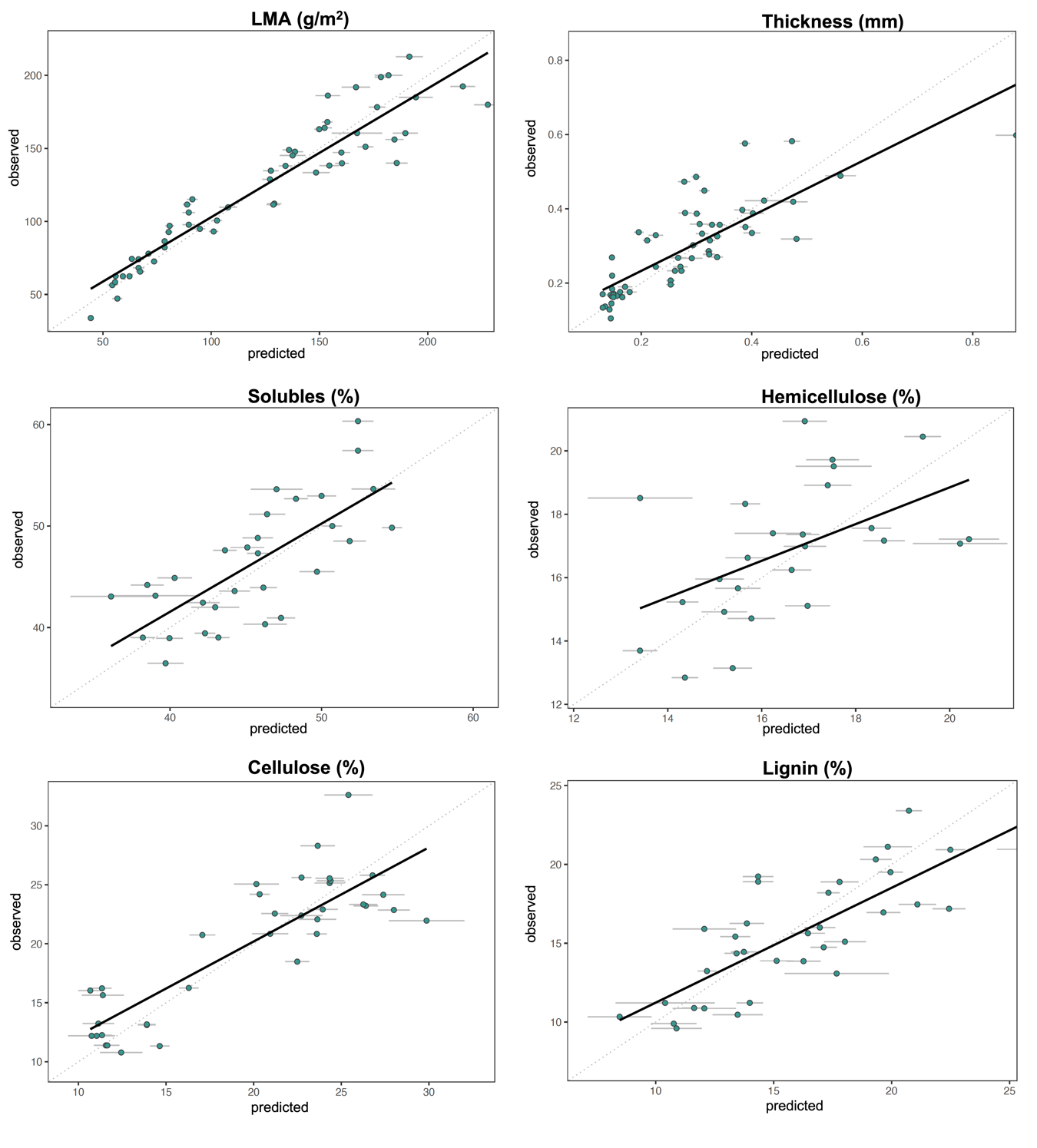

**Fig. S2 Internal validation results for LMA, Thickness, Solubles, Hemicellulose, Cellulose and Lignin predicted from dry spectra.** The error bars for each data point are 95% confidence intervals calculated from the distribution of predictions based on the ensemble of 1000 iterations.


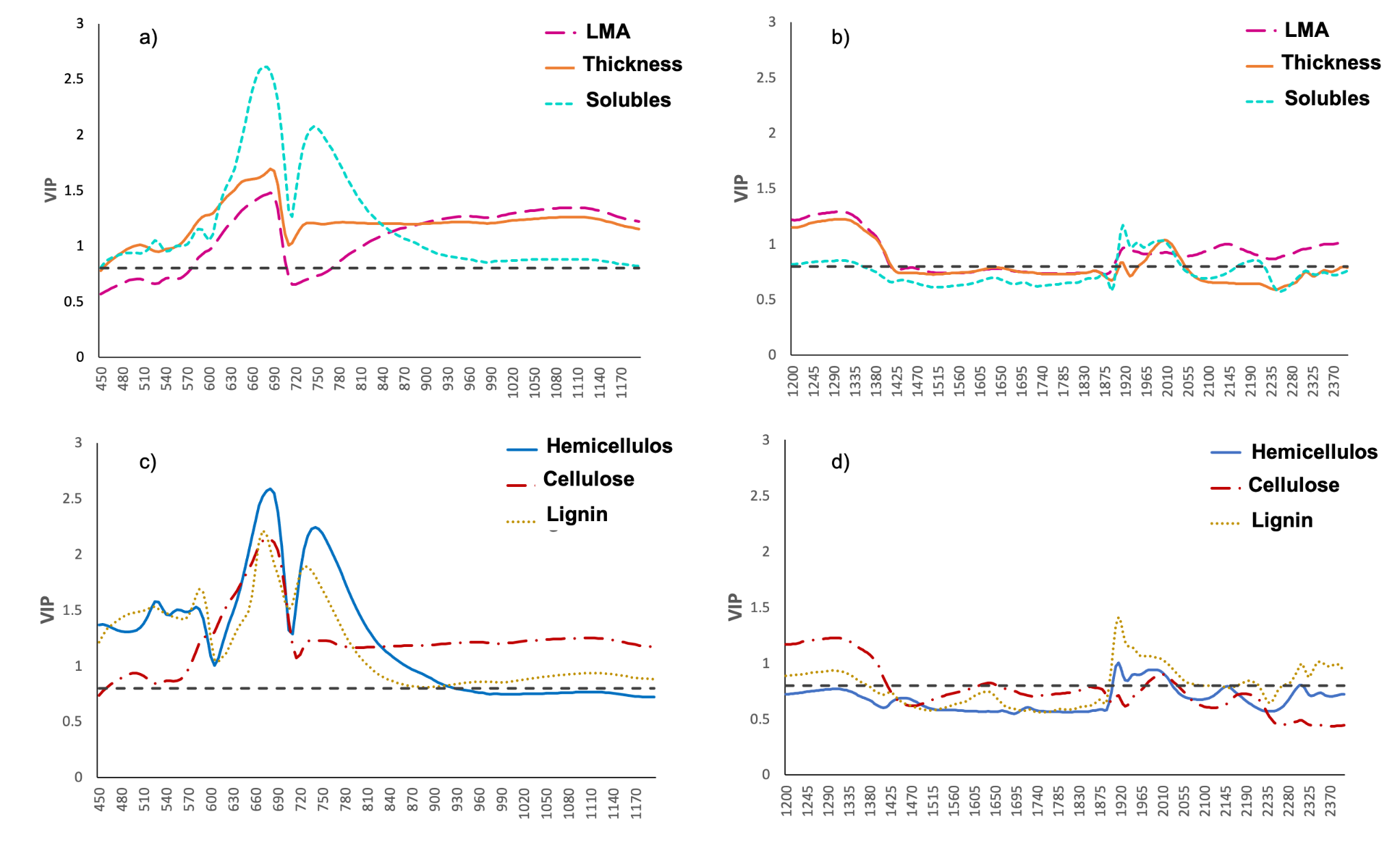


**Fig. S3. The variable importance in the projection (VIP) metric was calculated based on dry sample spectral data models for six traits.** **a**) and **c**) represent wavelengths from 400 to 1200 nm VIP, **b**) and **d**) 1200 to 2400 nm VIP. The dashed horizontal line at 0.8 represents a heuristic threshold for importance, as suggested by Burnett *et al.* (2021).


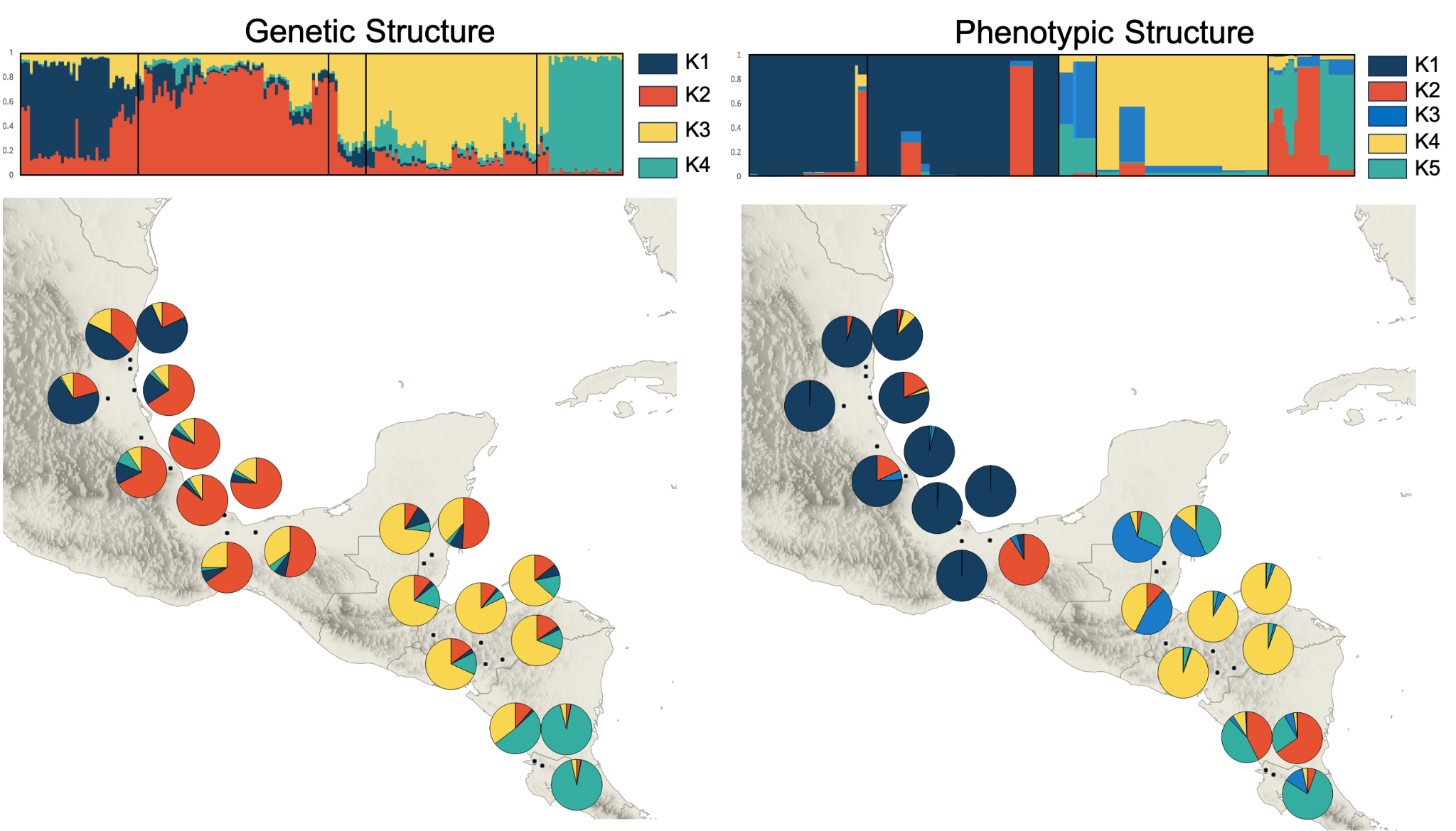


**Fig. S4**. **Spatial distribution of genetic and phenotypic variation in *Q. oleoides*. a)** Four genetic groups were identified using STRUCTURE (Pritchard 2000) from 123 individuals and **b)** five phenotypic groups identified by GENELAND (Guillot et al., 2009) using six spectrally derived leaf traits (leaf mass area, thickness, solubles, hemicellulose, cellulose, lignin) derived from dried leaf spectra from all individuals. The percentage assignment to genetic or phenotypic groups is represented at both the individual tree level (upper bar plots) and subpopulation level (pie charts).


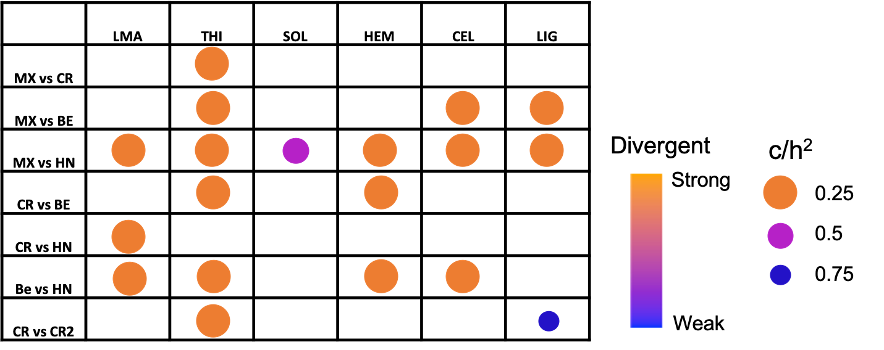


**Figure S5. *P*_ST_ comparisons among *Q. oleoides* genetic groups predicted using 11nSSR genetic markers. (MX) Mexico, (CR) Costa Rica, (CR2) Costa Rica two (Phenotypic group), (Be) Belize, (HND) Honduras.** Matrices represent *P*_ST_ values for six predicted traits from spectra plotted as a function of c/h^2^ values of 0.25 (orange), 0.5 (pink) and 0.75 (blue) in pairwise genetic groups. The optimal value of c/h^2^ at which the lower confidence limit of *P*_ST_ is higher than the upper confidence limit of *F*_ST_ was chosen as the critical value of c/h^2^ at which *P*_ST_ exceeds *F*_ST_. The lower this critical value, the more robust inferences of selection are to environmental effects. Predicted traits (LMA) Leaf mass area, (THI) thickness, (SOL) solubles, (HEM) hemicellulose, (CEL) cellulose, and (LIG) lignin.


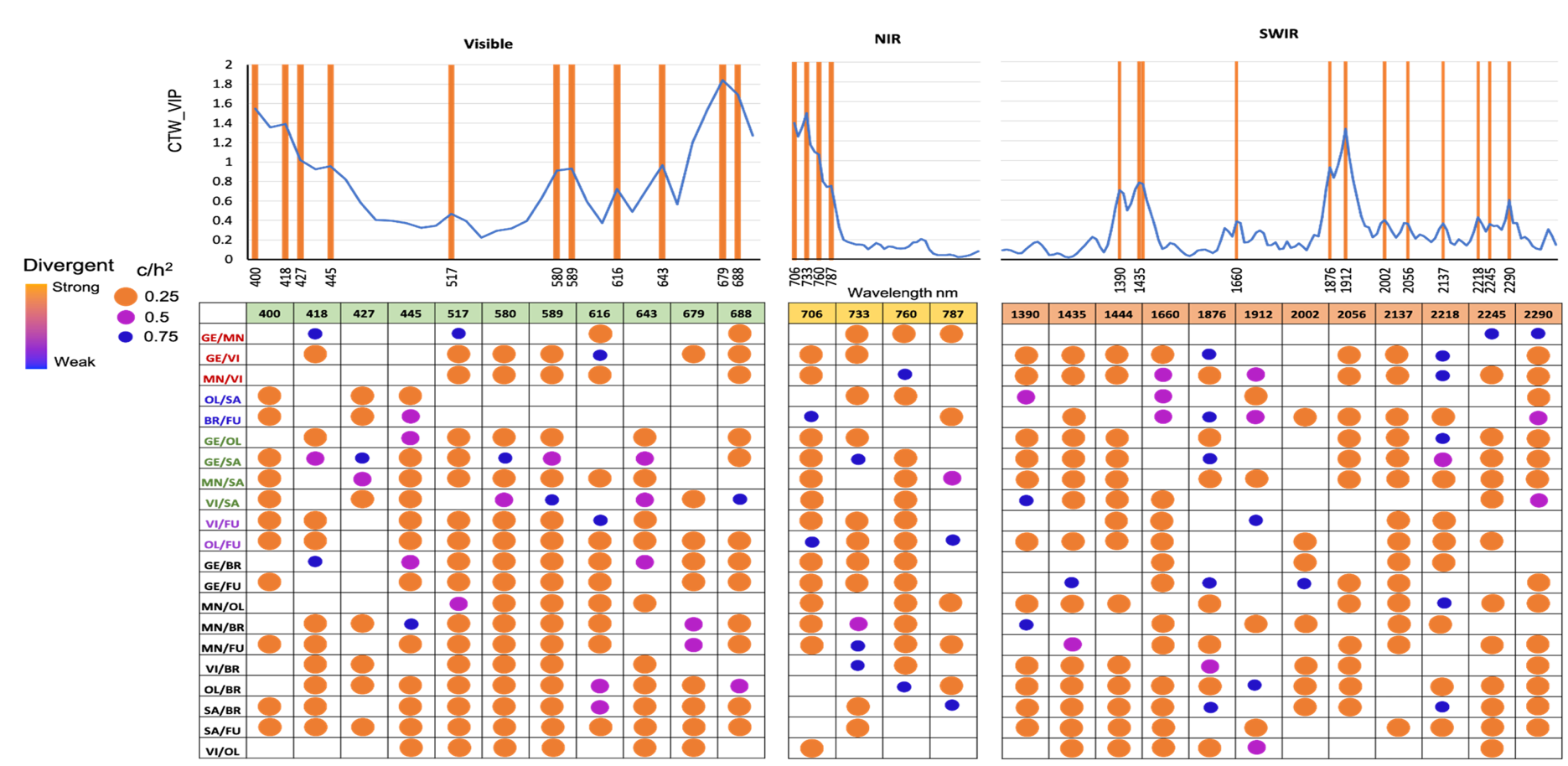


**Fig. S6. *P*_ST_ comparisons among species using: a) spectrally predicted traits (LMA: Leaf mass area; THI: thickness; SOL: solubles; HEM: hemicellulose; CEL: cellulose; LIG: lignin); b) spectral bands within the visible (VIS), near infrared (NIR) and short-wave infrared (SWIR) region with high importance (i.e., Variable Importance of Projection (VIP) in discriminating species using wavelet spectra.** Matrices represent *P*_ST_ plotted as a function of c/h^2^ values of 0.25 (orange), 0.5 (pink) and 0.75 (blue). The optimal value of c/h^2^ at which the lower confidence limit of *P*_ST_ is higher than the upper confidence limit of *F*_ST_ was chosen as the critical value of c/h^2^ at which *P*_ST_ exceeds *F*_ST_. The lower this critical value, the more robust inferences of selection are to environmental effects. Panel **a**) shows a simplified phylogenetic tree inferred from RADseq (min 20) data for 27 *Virentes* individuals using RAXML (Cavender-Bares et al., 2015) with the pairwise comparisons among species that were conducted using the six spectrally predicted traits. Colored lines represent sister relationships, historical introgression between specie pairs, and/or sympatric geographic associations within the *Virentes*: Red, sympatric sister species; Blue, sister but not sympatric species; Green, historically introgressing populations; Purple, parapatric species with introgression. Only phylogenetically and geographically meaningful pairwise comparisons are shown. Panel **b**) represents plotted VIP values obtained from the PLS-DA classification model using wavelet spectra: orange vertical lines represent wavelengths that were used as traits to calculate *P*_ST_ pairwise distances. Matrices are divided into VIS, NIR, and SWIR spectral regions. Contrary to panel **a**) all pairwise comparisons among species are shown, but name colors represent the same phylogenetic/geographic relations. GE= *Q. geminata*, MN= *Q. minima*, VI= *Q. virginiana*, SA= *Q. sagraeana*, OL= *Q. oleoides*, BR= *Q. brandegee*i, FU= *Q. fusiformis.*

**Table S5. Statistical associations between various plant traits, environmental variables, and spectral reflectance at specific wavelengths in three regions visible (VIS), near infrared (NIR), short wave infrared; SWIR), and Spectral INDEX.** The results include standard error (+/- SE) significance level (p < 0.05), coefficient of determination (R²), and adjusted R². Additionally, potential biochemical compounds linked to each spectral band are provided.

|  | **Trait** | **Environmental**  **Variable** | **+/- SE** | **p** | **R^2^** | **Adjusted R^2^** | **Potential Associated Trait**  **(Concentration/Reflectance)** |
| --- | --- | --- | --- | --- | --- | --- | --- |
| **Traits** | SOL **%** | Bio_18 | 0.15 | 0.001 | 0.58 | 0.56 |  |
|  | LIG **%** | Bio_6 | 0.11 | 0.04 | 0.03 | 0.29 |  |
|  |  | Bio_18 | 0.15 | 0.001 | 0.64 | 0.62 |  |
| **VIS** | 580nm | Bio_18 | 0.00 | 0.001 | 0.51 | 0.47 | Carotenoids, including β-carotene  (+/-) |
|  | 688nm | Bio_18 | 0.000 | 0.003 | 0.466 | 0.431 | Anthocyanins |
| **NIR** | 733nm | Bio_18 | 0.00 | 0.001 | 0.44 | 0.40 | Reflectance linked to leaf structure and mesophyll thickness.  (-/+) |
|  | 760nm | Bio_14 | 0.00 | 0.01 | 0.37 | 0.33 | Potential influence of leaf structural traits.  (-/+) |
|  | 787nm | Bio_8 | 0.00 | 0.04 | 0.25 | 0.20 | Leaf thickness, Leaf Mass Area (LMA),  (-/+) |
| **SWIR** | 1660nm | Bio_18 | 0.00 | 0.02 | 0.33 | 0.28 | Lignin and cellulose  (-/+) |
|  |  | Wetness index | 0.01 | 0.04 | 0.25 | 0.20 |  |
|  | 2050nm | Bio_8 | 0.00 | 0.001 | 0.47 | 0.43 | Cellulose, lignin (OH and CH bond absorptions)  (-/+) |
| **INDEX** | CCI | Bio_18 | -0.78 | 0.14 | 0.0005 | 0.68 |  |
|  | Chlorophyll | Bio_18 | -0.78 | 0.14 | 0.0005 | 0.68 |  |
|  | Chlorophyll | Bio_8 | -0.46 | 0.16 | 0.01 | 0.36 |  |
|  | ARI1 | Bio_18 | 0.80 | 0.15 | 0.00 | 0.64 |  |
|  | ARI1 | Bio_12 | 0.64 | 0.19 | 0.00 | 0.43 |  |
|  | ARI1 | Bio_8 | 0.54 | 0.15 | 0.00 | 0.45 |  |

**
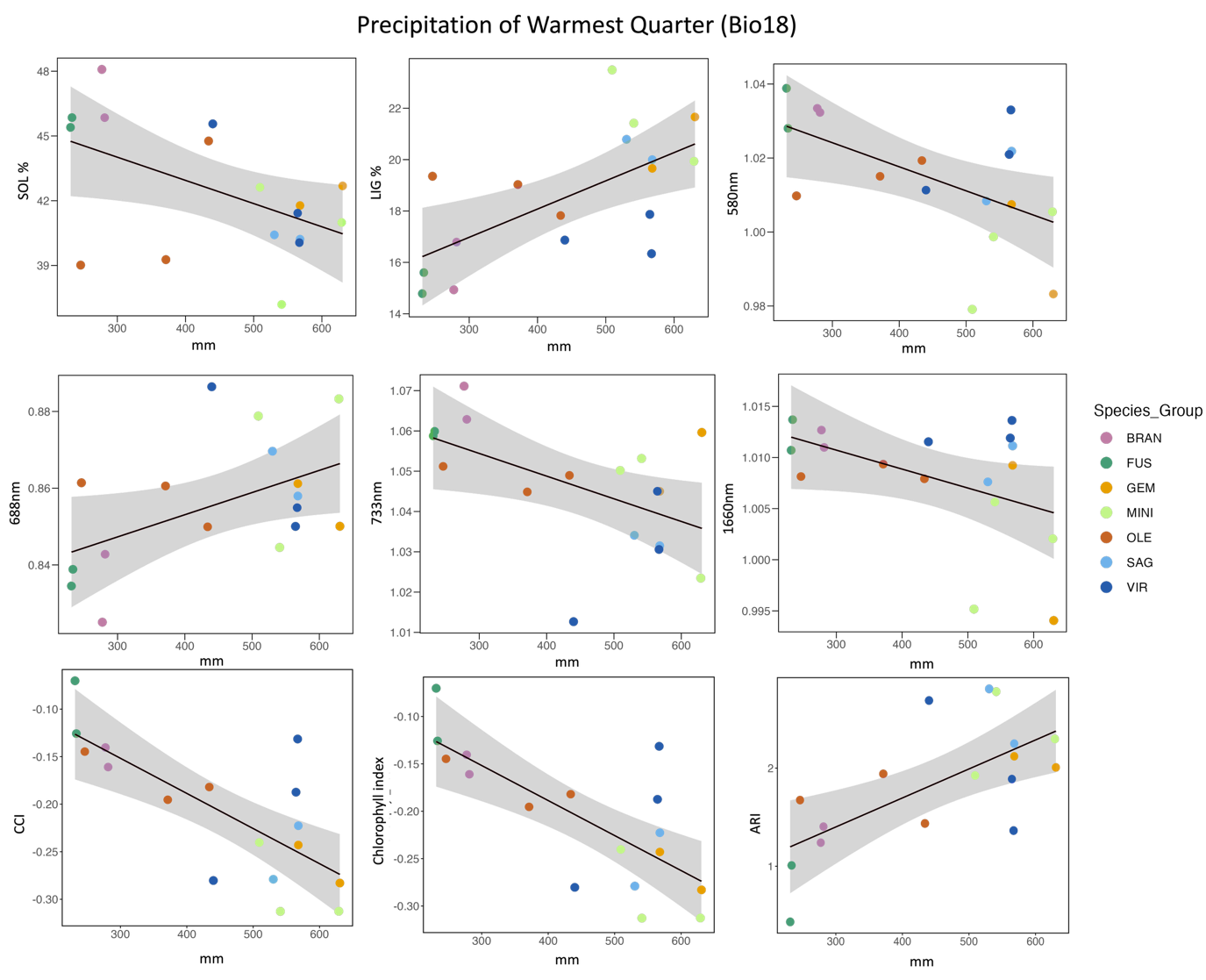
**

**
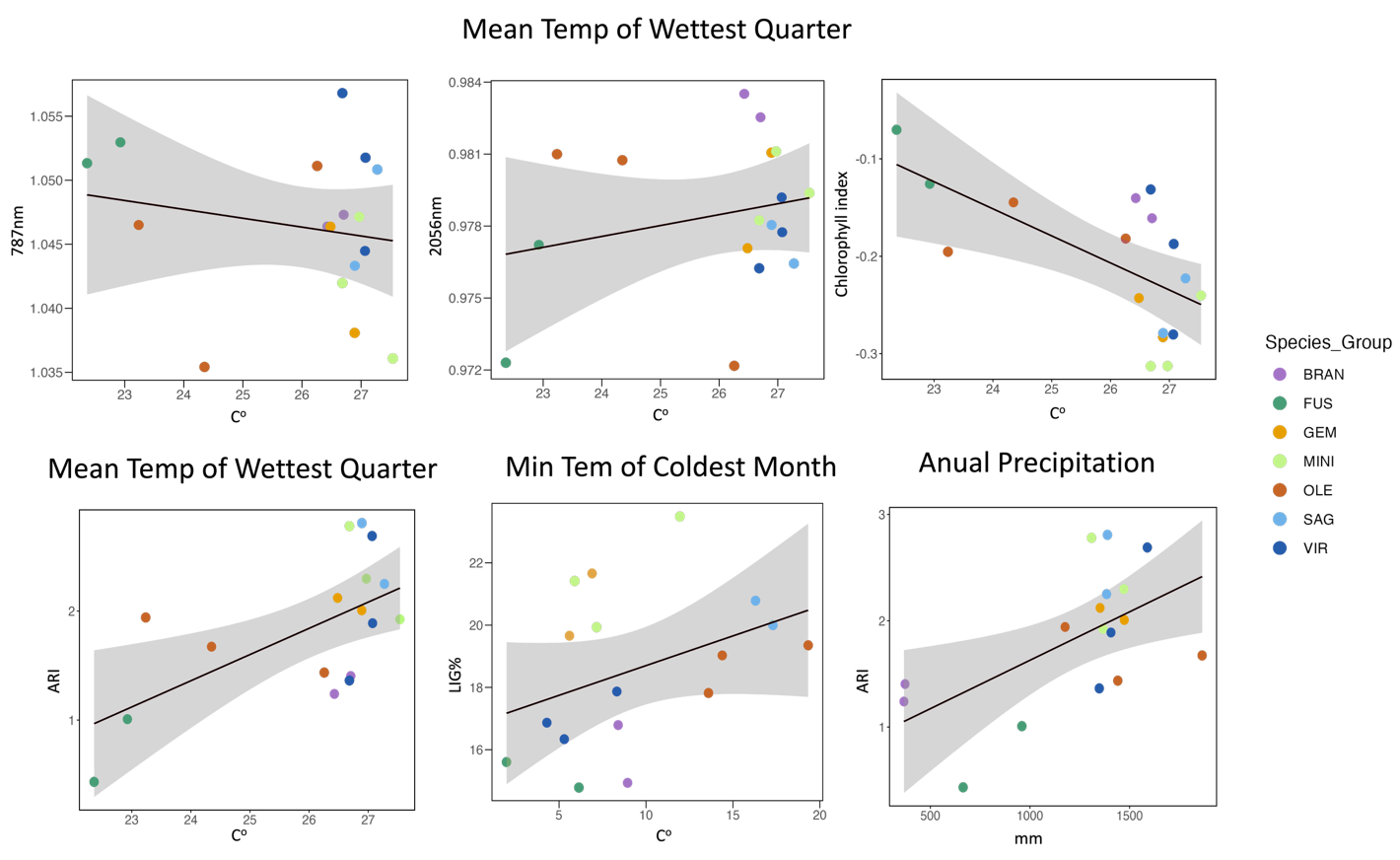
**

**Fig. S7. Spectrally predicted traits and selected wavelength relationships under different environmental conditions.** Phylogenetic generalized least squares (PGLS) models account for relatedness across species. Coefficients for all models are given in Supporting Information **Table S5.**

**Table S6. Phylogenetic signal calculated using Blomberg’s K (K), K_WN_ white noise and K_BM_ Brownian motion estimated for the seven species in *Quercus Virentes* phylogeny, where regions with significant signal *(p < 0.05) ns, no significative**

| Region | wavelength/trait | *K* | *p*_value K | ***p* (*K* > *K*_WN_)** |
| --- | --- | --- | --- | --- |
| VIS | 400 | 0.67 | ** | ** |
|  | 418 | 1.11 | ** | ** |
|  | 427 | 0.52 | ** | ** |
|  | 445 | 0.64 | ** | ** |
|  | 517 | 0.59 | ** | ** |
|  | 580 | 0.59 | ** | ** |
|  | 589 | 0.60 | ** | ** |
|  | 616 | 0.90 | ** | ** |
|  | 643 | 0.39 | ns | ** |
|  | 679 | 0.51 | ** | ** |
|  | 688 | 0.49 | ** | ** |
| NIR | 706 | 0.83 | ** | ** |
|  | 733 | 0.58 | ** | ** |
|  | 760 | 0.44 | ** | ** |
|  | 787 | 0.38 | ns | ** |
|  | 985 | 0.30 | ns | ** |
| SWIR | 1390 | 0.23 | ns | ** |
|  | 1432 | 0.21 | ns | ** |
|  | 1435 | 0.23 | ns | ** |
|  | 1444 | 0.28 | ns | ** |
|  | 1660 | 0.34 | ns | ** |
|  | 1876 | 0.16 | ns | ** |
|  | 1912 | 0.18 | ns | ** |
|  | 2056 | 0.26 | ns | ** |
|  | 2137 | 0.29 | ns | ** |
|  | 2218 | 0.21 | ns | ** |
|  | 2245 | 0.16 | ns | ** |
|  | 2290 | 0.14 | ns | ** |
| Traits | LMA | 0.24 | ns | ** |
|  | thickness | 0.25 | ns | ** |
|  | solubles | 0.69 | ** | ** |
|  | hemicellulose | 1.79 | ** | ** |
|  | cellulose | 0.30 | ns | ** |
|  | lignin | 0.92 | ** | ** |


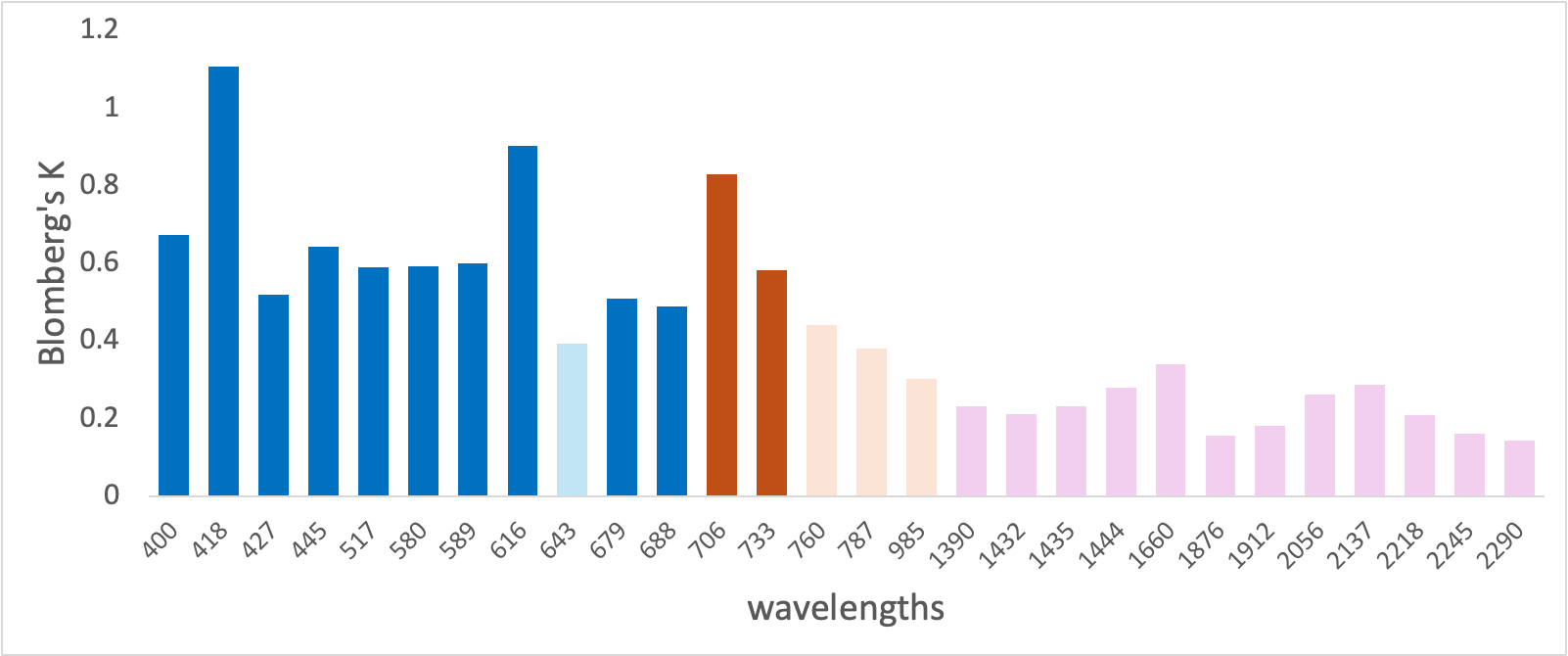


**Fig. S8 *Phylogenetic* signal detected in leaf spectra varies across wavelengths across *Quercus Virentes* species**. Phylogenetic signal calculated using Blomberg’s K (K) estimated, where regions with significant signal (p < 0.05) dark colors. Not significant values are colored with dim colors. Blue visible; red NIR; purple SWIR


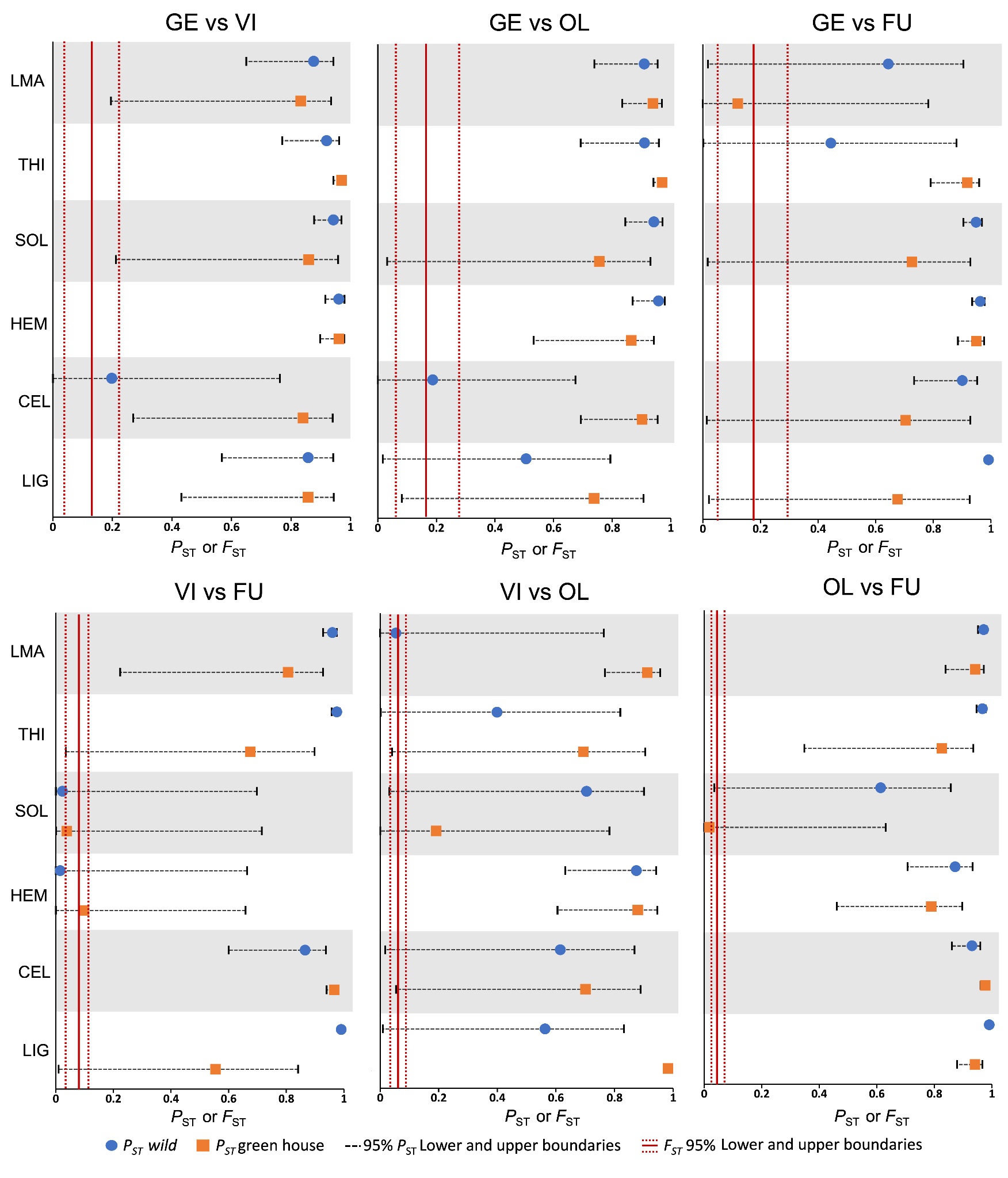


**Fig. S9. *P*_ST_ vs *F*_ST_ estimates and 95% confidence intervals from quantitative predicted traits among wild and greenhouse individuals for four species in *Quercus* section *Virentes*.** Graphs represented in colored blue circles (wild), and orange squares (greenhouse) mean *P*_ST_ values plotted as a function of c/h^2^ = 0.75. GE= *Q. geminate*, VI= *Q. virginiana*, OL= *Q. oleoides*, FU= *Q. fusiformis.* Predicted traits (LMA) Leaf mass area, (THI) thickness, (SOL) solubles, (HEM) hemicellulose, (CEL) cellulose, and (LIG) lignin.

**References**

Aitchison, J. (1986). The Statistical Analysis of Compositional Data. Springer Netherlands.

Brommer, J. E. (2011). Whither Pst? The approximation of Qst by Pst in evolutionary and conservation biology. Journal of Evolutionary Biology, 24(6), 1160–1168.

Burnett, A. C., Anderson, J., Davidson, K. J., Ely, K. S., Lamour, J., Li, Q., Morrison, B. D., Yang, D., Rogers, A., & Serbin, S. P. (2021). A best-practice guide to predicting plant traits from leaf-level hyperspectral data using partial least squares regression. Journal of Experimental Botany, 72(18), 6175–6189.

Cavender‐Bares, J., González‐Rodríguez, A., Eaton, D. A. R., Hipp, A. A. L., Beulke, A., & Manos, P. S. (2015). Phylogeny and biogeography of the American live oaks (*Quercus* subsection *Virentes*): a genomic and population genetics approach. Molecular Ecology, 24(14), 3668–3687.

Carrero C, Jerome D, Beckman E, Byrne A, Coombes A, Deng M, González Rodríguez A, Van Sam H, Khoo E, Nguyen N, Robiansyah I, Rodríguez Correra H, Sang J, Song Y-G, Strijk J, Sugau J, Sun W, Valencia-Ávalos S, Westwood M. 2020. *The Red List of Oaks 2020.*

Cruz-Nicolás, J., Giles-Pérez, G., González-Linares, E., Múgica-Gallart, J., Lira-Noriega, A., Gernandt, D. S., Eguiarte, L. E., & Jaramillo-Correa, J. P. (2019). Contrasting evolutionary processes drive morphological and genetic differentiation in a subtropical fir (Abies, Pinaceae) species complex. Botanical Journal of the Linnean Society. <https://doi.org/10.1093/botlinnean/boz077>

Denvir, A. & Westwood, M. (2016.) Quercus brandegeei, Encino Arroyo. The IUCN Red List of Threatened Species 2016: e.T30726A2795363. https://dx.doi.org/10.2305/IUCN.UK.2016- 3.RLTS. T30726A2795363.en

Earl, D. A., & vonHoldt, B. M. (2012). STRUCTURE HARVESTER: a website and program for visualizing STRUCTURE output and implementing the Evanno method. Conservation Genetics Resources, 4(2), 359–361.

Evanno, G., Regnaut, S., & Goudet, J. (2005). Detecting the number of clusters of individuals using the software structure: a simulation study. Molecular Ecology, 14(8), 2611–2620.

Hipp, A. L., Manos, P. S., Hahn, M., Avishai, M., Bodénès, C., Cavender-Bares, J., Crowl, A. A., Deng, M., Denk, T., Fitz-Gibbon, S., Gailing, O., González-Elizondo, M. S., González-Rodríguez, A., Grimm, G. W., Jiang, X.-L., Kremer, A., Lesur, I., McVay, J. D., Plomion, C., … Valencia-Avalos, S. (2020). Genomic landscape of the global oak phylogeny. New Phytologist, 226(4), 1198–1212.

Koehler, K., Center, A., & Cavender‐Bares, J. (2012). Evidence for a freezing tolerance–growth rate trade‐off in the live oaks (*Quercus* series *Virentes* ) across the tropical–temperate divide. New Phytologist, 193(3), 730–744.

Li, G., Yang, D., & Sun, S. (2008). Allometric relationships between lamina area, lamina mass and petiole mass of 93 temperate woody species vary with leaf habit, leaf form and altitude. Functional Ecology, 22(4), 557–564. https://doi.org/10.1111/j.1365-2435.2008.01407.x

Mevik, B., Wehrens, R. and Liland, K. H. (2020). pls:Partial Least Squares and Principal Component Regression. R package version 2.7-3. [https://CRAN.Rproject.org/package=pls](https://cran.rproject.org/package=pls)

Nei, M. (1972). Genetic distance between populations. *The American Naturalist, 106*(949), 283–292

Niklas, K. J., Cobb, E. D., & Spatz, H. (2009). Predicting the allometry of leaf surface area and dry mass. American Journal of Botany, 96(2), 531–536. https://doi.org/10.3732/ajb.0800250

Niklas, K. J., Cobb, E. D., & Spatz, H.-C. (2009). Predicting the allometry of leaf surface area and dry mass. *American Journal of Botany*, *96*(2), 531–536.

Ramírez-Valiente, J. A., & Cavender-Bares, J. (2017). Evolutionary trade-offs between drought resistance mechanisms across a precipitation gradient in a seasonally dry tropical oak (*Quercus* oleoides). Tree Physiology, 37(7), 889–901

Seeholzer, G. F., & Brumfield, R. T. (2018). Isolation by distance, not incipient ecological speciation, explains genetic differentiation in an Andean songbird (Aves: Furnariidae: Cranioleuca antisiensis, Line‐cheeked Spinetail) despite near threefold body size change across an environmental gradient. Molecular Ecology, 27(1), 279–296. https://doi.org/10.1111/mec.14429

Shi, P., Liu, M., Ratkowsky, D. A., Gielis, J., Su, J., Yu, X., Wang, P., Zhang, L., Lin, Z., & Schrader, J. (2019). Leaf area–length allometry and its implications in leaf shape evolution. Trees, 33(4), 1073–1085. https://doi.org/10.1007/s00468-019-01843-4

Teixeira, J. C., & Huber, C. D. (2021). The inflated significance of neutral genetic diversity in conservation genetics. Proceedings of the National Academy of Sciences, 118(10). https://doi.org/10.1073/pnas.2015096118

Wold, S., Sjöström, M., & Eriksson, L. (2001). PLS-regression: a basic tool of chemometrics. Chemometrics and Intelligent Laboratory Systems, 58(2), 109–130.
